# Supplementary material for: Process evaluation of the New Interventions for independence in Dementia Study (NIDUS) Family stream randomised controlled trial: protocol
Source: BMJ Open. 2022 Jun 8;12(6):e054613. doi: 10.1136/bmjopen-2021-054613 (PMC9185390; doi:10.1136/bmjopen-2021-054613)
Supplement: Supplementary data [file bmjopen-2021-054613supp001.pdf]

Appendix A

Alignment of planning, design and analysis of the NIDUS-Family process evaluation with MRC guidance.

Phase	MRC guideline recommendations (Moore et al, 2015)	Consideration of the recommendations for NIDUS-Family process evaluation
**Planning**	Define parameters of relationships of evaluators with intervention developers or implementers, balancing needs for good working relationships and independence; and how evaluators will inform implementation.	• Process evaluation led by a separate University.<br>• Evaluator is associate staff member at the trial University.<br>• NIDUS facilitators are employed through the intervention.<br>• Findings will inform the post-trial implementation strategy. They will not feed into the ongoing trial.
	Ensure the research team has the correct expertise, including, qualitative and quantitative research methods, and inter-disciplinary theoretical expertise.	Multi-disciplinary team includes expertise in psychology (ageing and behavioural change), old age psychiatry and dementia, neuropsychology, health service process evaluations, qualitative, quantitative, and mixed methods.
	Process and outcome evaluation team's degree of separation or integration:<br>• Oversight by a principal investigator.<br>• Good communication systems.<br>• Integration plans for process and outcome data agreed from the outset.	• Principal investigator has oversight over the NIDUS-Trial and is a subsidiary supervisor for evaluation lead.<br>•Evaluation is independent to the NIDUS-Family trial, but with weekly communication.<br>• Integration of process and outcome data will feed into the implementation study and strategy, but not into the trial.
**Designing**	Describe the intervention and its causal assumptions.	• The NIDUS-Family theory and causal assumptions are represented in a logic model (Figure 3).<br>• Section 1.1 describes the intervention, and 1.4 describes the causal assumptions
	• Identify questions by considering the intervention.<br>• Agree scientific and policy priority questions by considering the evidence for intervention assumptions.<br>• Consult with the evaluation team and policy/practice stakeholders.<br>• Identify previous process evaluations of similar interventions.	• The logic model informed the evaluation research questions.<br>• The multi-disciplinary team, including PPI, were consulted on the logic model.<br>• Relevant process evaluations were identified through a systematic review (PROSPERO ID: CRD42020221337).
	• Use quantitative methods to quantify key process variables and allow testing of pre-hypothesised	• Quantitative and qualitative methods will build upon one another to test, refine, and develop the NIDUS-Family

	mechanisms of impact and contextual moderators.<br>• Use qualitative methods to capture emerging changes in implementation, experiences of the intervention and unanticipated or complex causal pathways, and to generate new theory.<br>• Balance collection of data on key process variables from all sites or participants, with detailed case studies of purposively selected samples.<br>• Consider data collection at multiple time points to capture changes to the intervention over time.	logic model and emerging theory model (Figure 4).<br>• Quantitative methods will capture population level data on acceptability, reach, dose, attrition and secondary trial measures (approx. n=199). Quantitative observation data (approx. n=30) will enable detailed dyadic case-studies<br>• Qualitative interviews with purposively sampled dyads using GAS ratings (approx. N=30) will capture dyads experiences of receiving the intervention for case-studies and theme generation.<br>• Quantitative and qualitative methods will be matched on construct.<br>• Purposive sampling will recruit a sample representative of the trial population.<br>• Participants who withdraw will complete a questionnaire or an interview.<br>• Data collection at post 12-month follow-up for dyads and throughout for facilitators.
**Analysis**	Provide descriptive quantitative information on fidelity, dose and reach.	Fidelity: Fidelity checklist ratings for 20% of intervention-arm participants<br>Dose: number of sessions<br>Reach: Sites and locations<br>Attrition: Rate of withdrawal
	modelling of variations between participants or sites for factors such as fidelity or reach.	Contextual factors related to demographic data will be factored into data analysis and integration.
	Integrate quantitative process data into outcomes datasets, examining whether effects differ by implementation or pre-specified contextual moderators, and test hypothesised mediators.	Secondary trial data, dyadic observation fidelity checklist data, and acceptability ratings will be integrated to understand factors relating to high and low goal attainment.
	Collect and analyse qualitative data iteratively so that themes that emerge in early interviews can be explored in later ones.	Qualitative data collection and analysis will be carried out iteratively as dyads finish their 12-month follow-up. Emerging themes from earlier interviews will be explored in later interviews.
	quantitative and qualitative analyses build upon one another, with qualitative data used to explain	A two-stage integration approach will be used to merge the findings, initially at the

	quantitative findings, and quantitative data used to test hypotheses generated by qualitative data.	level of the dyad, then at the population level.
	Initially analyse and report qualitative process data prior to knowing trial outcomes to avoid biased interpretation.	Qualitative data will be collected and analysed before trial outcomes are known.
	Report whether process data are being used to generate hypotheses (analysis blind to trial outcomes), or for post-hoc explanation (analysis after trial outcomes are known).	Process data will be used to generate hypotheses, analysis will be blind to primary trial outcomes. Secondary outcomes will be analysed.

Note. Adapted from Moore et al (2015, p12)
