# Supplementary material for: Process evaluation of the New Interventions for independence in Dementia Study (NIDUS) Family stream randomised controlled trial: protocol
Source: BMJ Open. 2022 Jun 8;12(6):e054613. doi: 10.1136/bmjopen-2021-054613 (PMC9185390; doi:10.1136/bmjopen-2021-054613)
Supplement: Supplementary data [file bmjopen-2021-054613supp002.pdf]

Appendix B

Matching Quantitative and Qualitative Constructs Examples

<table>
<tr><th rowspan="3">Construct</th><th rowspan="3">Associated causal assumptions</th><th colspan="2">Quantitative questions
Method: Acceptability questionnaire and observation data collected through listening to video/audio recordings of dyads session

Variable: rate 1 Strongly disagree – 5 strongly agree)</th><th colspan="2">Qualitative questions
Method: Qualitative semi-structured interviews</th></tr>
<tr><th rowspan="2">Acceptability questionnaire

(Appendix F)</th><th rowspan="2">Observation checklist (Appendix E)</th><th>Dyad (Appendix C)</th><th>Facilitator (Appendix D)</th></tr>
<tr><th></th><th></th></tr>
<tr><td rowspan="4">Values and approaches</td><td>CA1.1</td><td></td><td>The facilitator promoted choice [for PLWD/ for Carer]</td><td>Do you feel you were able to contribute to the sessions?</td><td>Do you feel you promoted the dyad to have choice?</td></tr>
<tr><td>CA1.1, 1.2, 8</td><td>[myself/ the person I care for] had a good relationship with my facilitator</td><td>Discussions were respectful/ supportive [for PLWD/ for Carer] (allowing others to speak, actively listening, supporting their opinions, working as partners, discussing differing opinions calmly)</td><td>• Do you feel you were respected?
•Do you feel you built up a level of trust with your facilitator?
• Do you feel the relationship was mutual and reciprocal?</td><td>• Do you feel you built a sense of trust with the dyad?
• Do you feel you actively listened?
• Do you feel there was mutual respect between you and the dyad?</td></tr>
<tr><td>CA8</td><td></td><td>[PLWD/ Carer] had opportunities to ask questions.</td><td>How were your discussions in the sessions?</td><td>Who was involved in the discussion?</td></tr>
<tr><td>CA1.3, 8</td><td>[I/ the person I care for] contributed to decision making.</td><td>[PLWD/ Carer] contributed to decision making.</td><td>Did you feel involved in the decision-making?</td><td>Who was involved in decision-making?</td></tr>
</table>

	CA1.1	[I/ the person I care for] had opportunities for meaningful e ngagement.	[PLWD/ Carer] had opportunities for meaningful engagement *(a ble to actively participate, actively contribute ideas, skills or abilities)*	Do you feel you were able to actively participate?	Do you feel the dyad had opportunities for meaningful engagement?
	CA2.1		[facilitator/Car er] showed compassion *(di d they take time to bond, act with kindness, be encouraging, be polite)*	How did the facilitator make you feel?	How would you describe your persona in the sessions?
	CA2.2		[facilitator/PL WD/ Carer] explored risks [for PLWD/ for carer]	Did you discuss any possible risks?	Tell me about a risk you discussed and how you managed this (when setting tasks/ goals)?
	CA5	Goals were tailored to the [PLWD/ family member] nee ds.	The facilitator tailored [PLWD/ Carer] needs/goals/pl ans/activities/t asks.	• Do you feel the goals set reflected your needs/issues at the time?<br>• What plans, activities, tasks did you put in place to work towards your goals?	Once the goals were set can you talk through how you developed [plans/ activities/ actions] for the dyad to work towards their goals…
	CA1.3, 8		[facilitator/PL WD/ Carer] agreed (acknowledged) next steps (actions to follow the session)	Were you clear on activities between sessions?	*Who took accountability for actions?*
	CA8		The [PLWD/carer] acknowledged/ took ownership	Who took charge of doing the	Who took accountability for actions?

			for the actions/tasks s et.	activities/plan s?	
Goals and Strategies	CA6.3		Goals were discussed .	Tell me about your goals…	How did the [PLWD/carer] interact in the sessions?
	CA6.3	The modules helped [me/ the person I care for] work towards my goals...	Modules were discussed in line/ linked with the dyad's goals.	How did module [X] fit with/ affect your goals?	Talk me through how the modules worked for the dyad…
	CA6.3		[PLWD/ carer] engaged with the module/s...	How did you find the modules?	How do you feel the dyad engaged with the modules?
	CA6.3		Clear objectives/ next steps were set [for PLWD/Carer]	Who took charge of doing the activities/plan s?	Who took accountability for actions?
