# Supplementary material for: Process evaluation of the New Interventions for independence in Dementia Study (NIDUS) Family stream randomised controlled trial: protocol
Source: BMJ Open. 2022 Jun 8;12(6):e054613. doi: 10.1136/bmjopen-2021-054613 (PMC9185390; doi:10.1136/bmjopen-2021-054613)
Supplement: Supplementary data [file bmjopen-2021-054613supp003.pdf]

Appendix C

Semi-structured interviews for dyads

Interview Approach

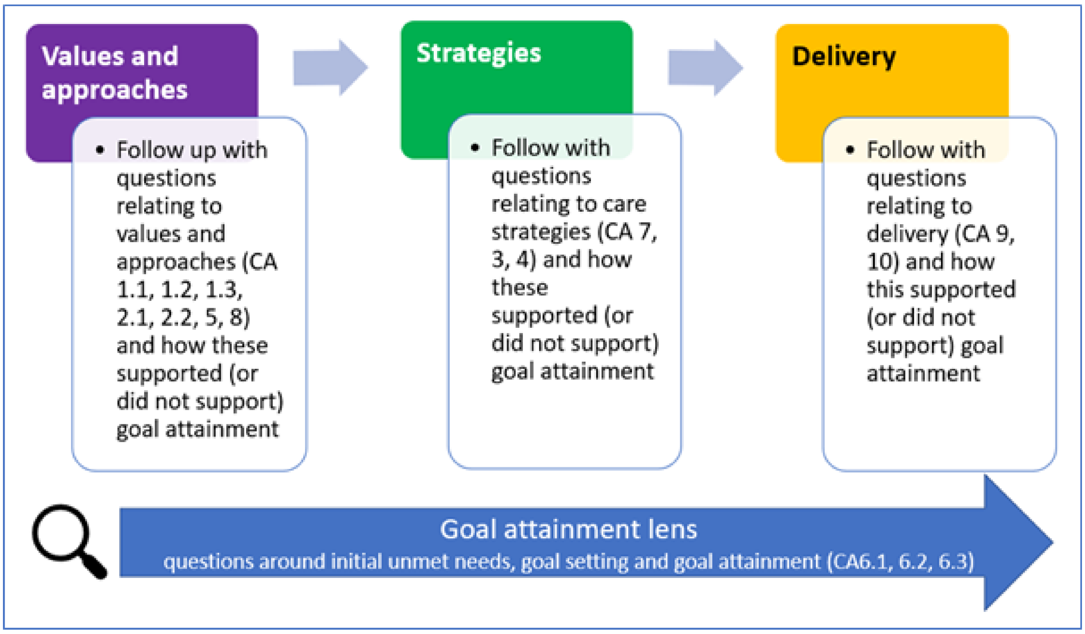

Semi-structured topic guide for dyads (PLWD and carer)

This will be used as a guide, a flexible approach will be adapted to capture any relevant information as well as unanticipated or unexpected pathways.

Introduction:

"Hello, I am XXXX and my role is XXXX. Thank you for your time today, I understand you have taken part in the NIDUS-Family intervention and I wanted to find out more about your experiences in receiving this. It would be interesting to explore your experiences of the NIDUS-Family trial, particularly in relation to your goals. There are no right or wrong answers, you are the experts. This interview will be audio-recorded and transcribed anonymously. Everything you say will be confidential and anonymised, any identifying information will be removed. You will be able to withdraw from this part of the study until the point of data analysis. Information from today will help us make sure any benefits of NIDUS-Family can be put into wider practice. Do you have any questions? If you are happy to continue, please can you give your consent (informed or by proxy) and I will start recording.".

Q1. Tell me about your experiences of NIDUS-Family… (where relevant ask PLWD first, then carer)

Prompts:

- How did you get involved with NIDUS-Family?
- What were your expectations of taking part in NIDUS-Family?

Q2. Tell me about the main things that have changed for you since finishing the NIDUS-Family intervention… *[ask PLWD and carer separately where possible]*

- How has that affected your day-to-day routine?
- How has that affected you?

Values and approaches

Q3. Tell me about your (PLWD and Carer) relationship?

- Overall:
  - *Day to day examples*
  - *Do you live together/ how often do you see each other?*
  - *Nature of visits?*
- NIDUS-Family specific
  - *How do you feel you worked together during the NIDUS sessions?*
  - *How were your discussions in the sessions?*
  - *Has your relationship changed since NIDUS-Family?*

Q4. Tell me about your relationship with the facilitator… (where relevant ask PLWD first, then carer)

  - *How did the facilitator make you feel?*
  - *Do you feel you were able to actively participate?*
  - *Do you feel you were able to contribute (ideas) to the sessions?*
  - *Did you feel involved in the decision-making?*
  - *Do you feel you built up a level of trust with your facilitator?*
  - *Do you feel the relationship was mutual and reciprocal?*
  - *Did you feel you partnered with the facilitator?*
  - *Do you feel you were respected?*
- Tell me how your relationship with your facilitator affected you attaining your goals?
- How did you feel about having the same facilitator throughout?

Q5. Tell me about your support network...

- informal – neighbours, friends, other family
- formal – services, resources
  - *Has this changed since starting/ finishing NIDUS-Family?*
  - *How did these (support/network/service use) changes affect your goals?*

Goal attainment

Q6. Tell me about your goals… (where relevant ask PLWD first, then carer)

Prompts:

- Do you feel the goals set reflected your needs/issues at the time?
  - *How did you discuss your needs?*
  - *Did you talk about your issues/needs?*
  - *Were they specific to you [PLWD and/or Carer]*
- Tell me more about how you decided on these goals?
  - *How were the goals set?*
  - *Who was involved in setting them?*
  - *How did that discussion go? Maybe we could use an example…*
- Do you feel your goals were achievable?
  - *[If not] Did you tell the facilitator you felt this at the time?*

- *Is this in hindsight?*
- Once you set your goals, tell me what you did next?
  - *How did you work towards your goals?*
  - *What plans, activities did you put in place?*
  - *Who came up with those plans?*
  - *Who took charge of doing the activites/plans?*
  - *Can you give me an example of something that helped?*
  - *Can you give me an example of something that may have got in your way?*
- Could you tell me why you scored your goals as (+2/ 0,-1,-2)?
- Knowing what you do now, is there anything you would have done differently?

Strategies

Q7. How did you find the modules (name modules *talk through one at a time*)?

- How did module [X] fit with your goals?
- How did module [X] affect your goals?
  - *What did you enjoy?*
  - *What did you not enjoy?*
- Were there any parts of the modules you liked?
- Were there any parts of the modules you didn't like?
- Is there anything you wish the modules had covered?

Q8. Before we finish, can I review the key points you mentioned about:

- Goal attainment
- Values and approaches
- Strategies
- Delivery

Q9. Is there anything else you would like to add?

Q10. Is there anything we haven't covered you feel is important?

Thank you for your time and for taking part today.
