# Supplementary material for: Process evaluation of the New Interventions for independence in Dementia Study (NIDUS) Family stream randomised controlled trial: protocol
Source: BMJ Open. 2022 Jun 8;12(6):e054613. doi: 10.1136/bmjopen-2021-054613 (PMC9185390; doi:10.1136/bmjopen-2021-054613)
Supplement: Supplementary data [file bmjopen-2021-054613supp005.pdf]

Appendix E

Observation/ Fidelity Checklist

For fidelity checklist only complete italicised sections. Complete all sections for process evaluation observations.

Process evaluation factors	Please rate<br>1 Strongly disagree, 2 disagree, 3 neither agree nor disagree, 4 agree, 5 strongly agree	Examples – include descriptive text or quotations to demonstrate related observations<br>*(Free text)*
**Values and approaches (CA1.1, 1.2, 1.3, 2.1, 2.2, 5, 8)**		
[facilitator/PLWD/ Carer] paid attention (are they focused) in the session. [Fidelity checklist]	*For PLWD:*<br>*For Carer:*<br>*Facilitator:*	
**[facilitator/PLWD/ Carer] were engaged (actively contributed, followed the discussions) in the session.**	For PLWD:<br>For Carer:<br>Facilitator:	
**[facilitator/PLWD/ Carer] contributed to discussions.**	For PLWD:<br>For Carer:<br>Facilitator:	
**The facilitator promoted choice (CA1.1).**	For PLWD:<br>For Carer:<br>Overall:	
**Discussions were respectful (allowing others to speak, supporting their opinions, working as partners, discussing differing opinions calmly - CA1.1, 1.2 8).**	Between PLWD and carer:<br>Between PLWD and facilitator:<br>Between carer and facilitator:<br>Overall	
**[PLWD/ Carer] had opportunities to ask questions.**	PLWD:<br>Carer:	
**[PLWD/ Carer] contributed to decision making (CA1.3)**	For PLWD:<br>For Carer:<br>Overall:	
**[PLWD/ Carer] had opportunities for meaningful engagement (able to actively participate, actively contribute ideas, skills or abilities) for PLWD (CA1.1)**	For PLWD:<br>For Carer:	
**[facilitator/Carer] showed compassion (did they take time to bond, act with kindness, be encouraging, be polite - CA2.1)?**	Facilitator to PLWD:<br>Carer to PLWD:<br>Facilitator to carer:	
**[facilitator/PLWD/ Carer] explored risks (CA2.2).**	For PLWD:<br>For Carer:<br>For facilitator:<br>Overall:	

**the facilitator tailored [PLWD/ Carer] needs/goals/plans/activities/tasks (CA8).**	For PLWD:<br>For Carer:<br>Overall:	Needs:<br>Goals:<br>Plans:<br>Activities:<br>Tasks:
**[facilitator/PLWD/ Carer] agreed (acknowledged) next steps (actions to follow the session).**	PLWD:<br>Carer:<br>Facilitator:	
**Did the [PLWD/carer] acknowledge/ take ownership for the actions/tasks set?**	PLWD:<br>Carer:	
**Goals (CA6.1, 6.2, 6.3) and Strategies (CA7, 3, 4)**		
**Goals were discussed.**		Which goals?
**Modules were discussed in line/ linked with the dyad's goals.**		Which modules?
**Clear objectives/ next steps were set [for PLWD/Carer].**	For PLWD:<br>For carer	
**The facilitator kept the [PLWD/Carer] focused on the module/goal.**		
**Overall**		
**The group was relaxed.**		
**The facilitator kept the [PLWD/carer] engaged in the session.**	PLWD:<br>Carer	
**The facilitator kept the [PLWD/ carer] focused on the manual/goals.**	PLWD:<br>Carer:	
**The facilitator kept the session to time.**		

**Any additional notes on the relationship dynamics between the:**<br>**• Facilitator and PLWD**<br>**• Facilitator and carer**<br>**• PLWD and carer (Free text)**

**Any additional notes on the session (Free text)**
Any additional notes on impact of COVID-19 on delivery (Free text) [Fidelity checklist]
Any additional notes for modifications to the intervention or facilitator training (Free text) [Fidelity checklist]
