# Supplementary material for: Process evaluation of the New Interventions for independence in Dementia Study (NIDUS) Family stream randomised controlled trial: protocol
Source: BMJ Open. 2022 Jun 8;12(6):e054613. doi: 10.1136/bmjopen-2021-054613 (PMC9185390; doi:10.1136/bmjopen-2021-054613)
Supplement: Supplementary data [file bmjopen-2021-054613supp006.pdf]

APPENDIX F

Acceptability Questionnaire for Family Carers

Family carer ID ------------------------- Person living with dementia ID: --------------------------

GAS scores: ---------------------------------

NIDUS-Family acceptability scale: Family carer (Please tick the box you feel is the most relevant answer related to the question, rating from strongly disagree to strongly agree)

Statement		Strongly disagree	Disagree	Neither agree nor disagree	Agree	Strongly agree
The intervention helped the person I care for.						
[I/ the person I care for] contributed to decision-making.	Myself (family carer)					
	The person I care for					
[I/ the person I care for] had opportunities for meaningful engagement *(able to actively participate, actively contribute ideas, skills or abilities)*	Myself (family carer)					
	The person I care for					
Goals were tailored to [my/ the person I care for] needs.	Myself (family carer)					
	The person I care for					
The modules helped [me/ the person I care for] work towards my goals.	Myself (family carer)					
	The person I care for					
[myself/ the person I care for] had a good relationship with my facilitator.	Myself (family carer)					
	The person I care for					
The intervention helped improve my relationship with the person I care for.						

What feedback do you have for us about your experiences of receiving the NIDUS-Family intervention?

………

…… ***[Please turn over for more space]***
