# Supplementary material for: Process evaluation of the New Interventions for independence in Dementia Study (NIDUS) Family stream randomised controlled trial: protocol
Source: BMJ Open. 2022 Jun 8;12(6):e054613. doi: 10.1136/bmjopen-2021-054613 (PMC9185390; doi:10.1136/bmjopen-2021-054613)
Supplement: Supplementary data [file bmjopen-2021-054613supp007.pdf]

Appendix G

Withdrawal Questionnaire

Family carer ID -------------------------

Person living with dementia ID: ---------------------------

**What was the reason(s) you withdrew?**
**Would you do anything differently?**
**What would you change?**

**Any other comments**
