# Supplementary material for: Process evaluation of the New Interventions for independence in Dementia Study (NIDUS) Family stream randomised controlled trial: protocol
Source: BMJ Open. 2022 Jun 8;12(6):e054613. doi: 10.1136/bmjopen-2021-054613 (PMC9185390; doi:10.1136/bmjopen-2021-054613)
Supplement: Author's manuscript [file bmjopen-2021-054613.draft_revisions.pdf]

BMJ Open

BMJ Open is committed to open peer review. As part of this commitment we make the peer review history of every article we publish publicly available.

When an article is published we post the peer reviewers' comments and the authors' responses online. We also post the versions of the paper that were used during peer review. These are the versions that the peer review comments apply to.

The versions of the paper that follow are the versions that were submitted during the peer review process. They are not the versions of record or the final published versions. They should not be cited or distributed as the published version of this manuscript.

BMJ Open is an open access journal and the full, final, typeset and author-corrected version of record of the manuscript is available on our site with no access controls, subscription charges or pay-per-view fees (http://bmjopen.bmj.com).

If you have any questions on BMJ Open's open peer review process please email info.bmjopen@bmj.com

BMJ Open

Process evaluation of the New Interventions for independence in Dementia Study (NIDUS) Family stream randomised controlled trial: Protocol

Journal:	*BMJ Open*
Manuscript ID	bmjopen-2021-054613
Article Type:	Protocol
Date Submitted by the Author:	21-Jun-2021

Complete List of Authors:	Wyman, Danielle; Anglia Ruskin University - Cambridge Campus, Faculty of Science and Engineering<br>Butler, Laurie; Anglia Ruskin University - Cambridge Campus, Psychology<br>Cooper, Claudia; University College London, Psychiatry of Older Age<br>Bright, Peter; Anglia Ruskin University, Psychology<br>Morgan-Trimmer, Sarah; University of Exeter Medical School, Institute of Health Research<br>Barber, Julie; UCL, Statistical Science

Keywords:	Dementia < NEUROLOGY, Old age psychiatry < PSYCHIATRY, Clinical trials < THERAPEUTICS

SCHOLARONE™
Manuscripts

TITLE PAGE

TITLE: Process evaluation of the New Interventions for independence in Dementia Study (NIDUS) Family stream randomised controlled trial: Protocol

Corresponding author

Full Name: Danielle Laura Wyman

Address: 8 Wren Close, Ringwood, BH24 3RF

Email: dlw136@pgr.aru.ac.uk

Co-authors

Professor Laurie Butler, Psychology, Anglia Ruskin University, Cambridge.

Professor Claudia Cooper, Psychiatry of Older Age, University College London, London.

Professor Peter Bright, Psychology, Anglia Ruskin University, Cambridge

Dr Sarah Morgan-Trimmer, Institute of Health Research, University of Exeter.

Dr Julie Barber, Statistical Science, University College London.

Total word count: 4,000

Keywords: Process evaluation, dementia, quality of life,

ABSTRACT:

Introduction: NIDUS-Family is an Alzheimer's Society funded new manualised, multimodal psychosocial intervention to support People Living with Dementia (PLWD) to achieve goals that they and their family carers set, towards living as independently and as well as possible at home for longer. This process evaluation will be embedded within the NIDUS-Family Randomised Controlled Trial intervention-arm (n=199), testing how the intervention influences change, as measured by goal attainment. The evaluation will test, refine, and develop the NIDUS-Family theoretical model, associated causal assumptions and logic model to identify key mechanisms of impact, implementation and contextual factors influencing the intervention's effectiveness. Findings will inform how the program is implemented in practice.

Methods and analysis: The process evaluation will be theory-driven and apply a convergent mixed-methods design. Dyads (PLWD and family carer) will be purposively sampled based on high or low Goal Attainment Scaling scores (trial primary outcome). Qualitative interviews with dyads (approx. n=30) and their respective facilitators will explore their experiences of receiving and delivering the intervention. Interviews will be iteratively thematically analysed. *Matching* observational quantitative data will be collected concurrently from video and/or audio recordings of NIDUS-Family dyad trial sessions. Further quantitative data will be collected through an acceptability questionnaire for all intervention-arm dyads (n=199). Mixed-method integration will use an interactive analysis strategy, considering qualitative and quantitative findings through mixed-method matrix for dyadic level 'case-studies', and a joint display for 'population' level analysis and interpretation.

Ethics and dissemination: This work is carried out within the UCL Alzheimer's Society Centre of Excellence for Independence at home, NIDUS (New Interventions in Dementia Study) programme (Alzheimer's Society Centre of Excellence grant 330). NIDUS-Family RCT has NRES approval (reference: 19/LO/1667).

For peer review only - http://bmjopen.bmj.com/site/about/guidelines.xhtml

Findings will be disseminated through publications and conferences, and as recommendations for the implementation study and strategy.

TRIAL REGISTRATION DETAILS: Information on the NIDUS-Family RCT is available on the clinical trials register http://www.isrctn.com/ISRCTN11425138

STRENGTHS AND LIMITATIONS OF THIS STUDY

• This evaluation will place people living with dementia and their family carer as experts to inform how NIDUS-Family is implemented in practice.

• This evaluation will use a convergent mixed-methods design grounded within a theory-informed logic model to understand the mechanisms of impact, implementation and contextual factors that influence how the NIDUS-Family intervention works.

• Data analysis, integration and interpretation at an individual dyadic case-studies level using mixed-method matrix, and a population level using joint display, will provide a greater depth of understanding of how NIDUS-Family works.

• The evaluation will reflect and represent the geographical and cultural diversity of the NIDUS-Family trial, which is unlikely to be fully reflective of the underlying population.

• The findings of the process evaluation will directly inform the strategy for the implementation of NIDUS-Family into routine practice, if effectiveness is demonstrated.

For peer review only

For peer review only - http://bmjopen.bmj.com/site/about/guidelines.xhtml

1 INTRODUCTION

Dementia is a syndrome affecting multiple aspects of a person's cognitive function.[1] Currently an estimated 885,000 people are living with dementia in the UK, and this number is predicted to increase to over 1.2 million by 2030.[2] Approximately two thirds of people with dementia are living in their own homes,[3] and wish to remain doing so as independently as possible.[4]

Challenging or distressing behaviours associated with dementia can lead to family carer stress, poor relationships with home care services, poor self-care and home safety risks, and are common reasons why people living with dementia move to a care home.[4] Government policy emphasises the importance of reducing transitions of people living with dementia into 24-hour care.[2] To help achieve this, it advocates personalised support and adaptations to help people living with dementia to retain their independence.[5] Currently no interventions consistently demonstrate improvements to people living with dementia's life quality, functioning, or the time they can stay living at home in the UK.[6,7] Therefore, there is a need to establish an evidence base for interventions in improving personalised support, adaptations, independence, and quality of life of people living with dementia.

1.1 The New Interventions for Independence in Dementia Study (NIDUS): Family

The NIDUS-Family program is a new manualised, multimodal psychosocial intervention to support people living with dementia to live independently at home for as long, and as well as possible. The intervention focuses on behavioural change, and aims to promote living with quality of life, choice, autonomy, dignity and as independently as possible.[8] The trial's primary objectives are to evaluate the effect of NIDUS-Family and routine care, relative to routine care alone at 12-month follow up, on goal attainment as measured by family-carer rated Goal Attainment Scaling (GAS) scores, and its cost-effectiveness. Secondary outcomes will measure activities of daily living, quality of life, neuropsychiatric behaviours, apathy, anxiety and depression, and service receipt.

The NIDUS-Family intervention is founded upon several theoretical principles (Figure 1).

Figure 1.

NIDUS Theoretical Model of Independence at Home

Note. Lord et al (2020)

The NIDUS-Family intervention will recruit 297 participants with a diagnosis of dementia, living in their own home and their regular family carer, who is in at least weekly (including remote) contact. Channels of recruitment include clinicians and research nurses working in NHS trust memory clinics/ older adult mental health services and GP practices based in London, Bradford, Humber, Leeds, Oxfordshire, Buckinghamshire, Berkshire, Kent and Surrey. Participants will also be recruited directly from the recruitment database Join Dementia Research. Randomisation will be blocked and stratified by site using a 2:1 intervention: routine care allocation ratio. Consent processes, outcome assessments and intervention delivery will be conducted over 12-months in the participant's own home, at the offices of the recruiting facilitator or via telephone or video call, depending on individual participant preference and COVID-19 restrictions.

The participants randomised to the active intervention group (n=199) will receive between six-to-eight manualised sessions within the first six months. NIDUS-Family aims to support people with dementia and their family carers (a dyad) to address personalised goals aligned to living as well as possible at home. The manualised sessions will be tailored to each participant dyad depending on their preferences and needs and all are delivered by the same facilitator where possible, and audio recorded. The facilitators will be trained on how to deliver the intervention and supervised throughout by a clinical psychologist. In session one, to create a bespoke support programme, the

For peer review only - http://bmjopen.bmj.com/site/about/guidelines.xhtml

facilitator will explore the dyads' identified goals and map them onto the intervention's manualised modules. They will also explore their support networks and identify gaps, sign-posting participants to existing resources and services. The modules utilise components of behavioural management, carer support, psychoeducation, training carers in communication and coping skills, and environmental adaptations.[6] The manualised modules include information and strategies addressing:

- Accepting care, arranging, and planning for the future
- Communicating with people living with dementia, family, and professionals
- Managing behaviours and challenging behaviours
- Managing physical health conditions
- Exercise, activity, and mobility
- Managing low mood, anxiety, and apathy
- Carer wellbeing and support
- Safety, environment, telecare, strategies, supporting functioning at home
- Relaxation and stress management strategies
- Sleep and diet

During the final manualised session, the researcher and participant will put together an action plan based on strategies that have worked. Following this, one-to-two monthly telephone follow-up calls will be conducted with the participants up to 1 year from baseline. The follow-up calls will offer support and guidance on implementing their action plan and troubleshoot any problems.

The NIDUS-Family intervention can be defined as a complex intervention due to its multiple interacting components, including the personalised and tailored approach, the dyadic relationship, and the differing contexts such as living arrangements, within which the programme is implemented.

1.2 Process evaluation of the NIDUS-Family intervention

Process evaluations aim to provide a detailed understanding of an intervention to inform policy and/or implementation into practice. The Medical Research Council (MRC) guidance [9] recommends examining aspects of the intervention including *"implementation (the structures, resources and processes through which delivery is achieved, and the quantity and quality of what is delivered), mechanisms of impact (how the intervention activities, and participants' interactions with them, trigger change), and context (how external factors influence the delivery and functioning of interventions)"* [9, p10].

This process evaluation will use the MRC's systematic approach for planning, design, analysis, and reporting (Appendix A shows alignment to the guidance).[9]

1.3 Process evaluation aims and objectives

As recommended by the MRC guidelines, this process evaluation will apply a theory-driven approach to respond to the research question: how does the NIDUS-Family intervention influence goal attainment?

We will explore how the hypothesised causal chains interact, to test and generate theory about how the NIDUS-Family intervention influences change through:

1) Evaluating the mechanisms of impact, implementation, and contextual factors comprising the NIDUS-Family intervention, with a primary focus on factors relating to goal attainment.

For peer review only - http://bmjopen.bmj.com/site/about/guidelines.xhtml

2) Identifying which mechanisms, implementation and contextual factors are essential for influencing the effectiveness of the NIDUS-Family intervention.

1.4 Theoretical basis

The NIDUS theoretical model (Figure 1), and its underpinning theories [6] directly informed the development of the hypothesised NIDUS-Family intervention causal assumptions (Figure 2).

Figure 2.

Hypothesised NIDUS-Family Causal Assumptions

Note. Derived from NIDUS Theoretical Model of Independence at Home (Figure 1)

The NIDUS-Family logic model (Figure 3) in turn clarifies how the NIDUS-Family intervention is designed to realise its intended outcomes and overlays the related causal assumption (CA) pathways for goal attainment, values and approaches, strategies, and delivery.

Figure 3.

NIDUS-Family Logic Model

The logic model (Figure 3) represents how NIDUS-Family helps dyads to identify three-to-five unmet needs related to living for longer at home. These are turned into specific, measurable, attainable, relevant, and time-bound (SMART) goals. The personalised goals are then mapped onto the NIDUS-Family modules, which use environmental, psychological, and occupational therapy strategies to reduce disability from behavioural or functional impairments as far as possible. The dyad attends six to eight sessions with a facilitator to work through the modules, bringing together old and new strategies to formulate a final action plan for dyads to follow to help them attain their goals. The intended outcomes for dyads are to improve communication, increase positive shared activities and improve their overall dyadic relationship. By attaining their goals, their unmet needs will now be met, leading to improved quality of life, wellbeing and the PLWD living at home for longer.

This process evaluation will evaluate how the pathways delineated in the logic model (Figure 3) work in practice. The NIDUS-Family intervention aims to aid dyads (PLWD and family carers) to identify and prioritise their needs and goals related to the PLWD living well at home for longer. It will test the emerging theory of change for attainment of dyadic goals (Figure 4) which represents how the core theoretical principles and casual assumptions derived from the logic model influence behavioural change through goal attainment and posits "*NIDUS-Family supports dyads to attain their goals through applying values and approaches, and strategies, supported by delivery through a single point of contact, and consistent joined up care.*" Focusing on dyads with high (2) and low (0 or below) 12-month carer-rated GAS scores will identify how the intervention works and enable theory development and refinement.

Figure 4.

Emerging Theoretical Model of Change for Attainment of Dyadic Goals through NIDUS-Family Intervention (with associated causal assumptions)

2. Methods

For peer review only - http://bmjopen.bmj.com/site/about/guidelines.xhtml

2.1 Design.

A pragmatic paradigm—creating shared meaning and joint action [10]—will underpin the methodology to understand how the NIDUS-Family intervention works. To test, explore, refine, and develop the emerging NIDUS-Family theory of change (Figure 4) for dyadic goal attainment, and associated hypothesised causal assumptions, a convergent mixed-methods design will be applied (Figure 5).[9, 11] This design will integrate qualitative and quantitative data to help gain a more complete understanding of how NIDUS-Family, if effective, influences behavioural, lifestyle and environmental change to enable goal attainment. Qualitative and quantitative questions will be matched on the emerging theory of change constructs for values and approaches, strategies and delivery through a lens of goal attainment (Appendix B shows matched constructs).[12]

Figure 5.

Mixed-Methods Convergent Design

Dyadic-level data refers to the data collected from purposively sampled dyads—for high (2) and low (0 or below) 12-month carer-rated GAS scores—from the intervention-arm who attend qualitative semi-structured interviews (approx. n=30). Qualitative dyadic-level data (dyadic interviews, facilitator interviews, observational data, facilitator session notes, and researcher reflexive field notes) will be collected concurrently and thematically analysed iteratively. Quantitative observational data will also be collected concurrently, analysed, and used descriptively. Quantitative secondary trial data will be extracted from the trial database after it is finalised and locked, and used descriptively. Dyadic-level data will be triangulated using a mixed-method matrix to help identify trends and patterns. Collection, analysis, and integration of data at a dyadic-level, will help us to test the causal pathways delineated in the emerging theory (Figure, 4) to identify which theoretical values and approaches, and strategies are key to influencing goal attainment.

Trial population-level data refers to all dyads in the intervention-arm (approx. n=199). Qualitative acceptability data will be collected iteratively and thematically analysed. Quantitative acceptability data will be collected and analysed descriptively. Trial data for dose, reach, fidelity, and attrition will be extracted from the final trial database after it is locked and used descriptively. Trial population-level data will be integrated with the dyadic-level findings using a joint-display. This will help us draw inferences and interpretations of the essential values and approaches, and strategies to understand how NIDUS-Family works and help us develop, refine, and consolidate the emerging theoretical model of change and logic model.

2.3 Patient and Public Involvement

NIDUS-Family intervention stakeholders and the Patient and Public Involvement (PPI) group were consulted in the development of the NIDUS-Family logic model.

2.3 Sampling

For dyadic-level data, dyads will be purposively sampled using quantitative primary measure trial data for 12-month follow-up family-carer rated GAS scores. Dyads with high goal attainment (a score of +2), and dyads with low goal attainment (a score of 0 or below) scores will be invited to interview and their recorded trial sessions (minimum one where available) will be observed through watching/listening to recordings. Dyads' respective facilitators will be invited to interview. To ensure sufficient conceptual depth, the conceptual depth scale[14] based on range, complexity, subtlety, resonance, and validity, will be applied to guide sample size for the number of dyads to be interviewed (approx. 10% N=30). Facilitators will be invited to a second interview when sufficient conceptual depth for dyads is reached to capture any data for subsequently sampled dyads they facilitated. Sampling for high and low 12-month carer-rated GAS scores will help us to understand

For peer review only - http://bmjopen.bmj.com/site/about/guidelines.xhtml

what influences high scoring dyads to attain their goals, and why low scoring dyads do not attain their goals. This will help us to explore and identify the causal factors which contribute to people benefiting from the intervention.

For trial population-level data, all carers from the intervention-arm will be sent/ invited to complete via telephone call, an acceptability questionnaire at 12-month follow up. Relevant trial data for dose, reach, and attrition will be collected for all intervention-arm dyads. Data will be extracted from the final locked trial database after completion and analysed descriptively. This will help us to understand how the NIDUS-Family intervention influences goal attainment at the trial population level. Fidelity checklists will be applied to a sample of 20% of the intervention-arms transcribed trial session audio/video recordings.

2.4 Data collection

For dyadic-level data collection (intervention-arm dyads sampled for high (+2) and low (0 or below) carer-rated GAS scores at 12-month follow-up) we will collect:

- Qualitative semi-structured Interviews. Purposively sampled dyads, and their facilitator will be invited to separate interviews. Dyad interviews and facilitator interviews will be audio recorded, anonymised with pseudonyms, transcribed verbatim and uploaded onto NVivo 12 to manage the data analysis process.
    - The dyad semi-structured qualitative interview (see Appendix C for topic guide) will explore their experiences of how the NIDUS-Family approaches and values, and strategies influenced them in attaining their goals for high GAS scores (2+), and why low GAS scores (0 or below) had no change or did not attain their goals.
    - The semi-structured, qualitative interview with facilitators (see Appendix D for topic guide) will explore key factors they feel influenced dyadic goal attainment for the dyad(s) selected to whom they delivered the intervention, as well as their overall experiences of facilitating NIDUS-Family. Any novel data relevant to subsequent sampled dyads will be explored with the respective facilitator in a second interview when sufficient conceptual depth for dyads has been captured.
- Observational data for purposively sampled dyads attending interview. The evaluator will listen to the dyads' recorded trial sessions (minimum one session per dyad where available). Qualitative (aligned to 'free-text' sections) and quantitative (aligned to Likert scale ratings) data relating to the emerging theory will be captured in an observation checklist (Appendix E). To ensure validity, a second researcher will independently complete the observation checklist for a minimum of 10% of observed sessions and these observations may be drawn upon in the facilitator and dyad interviews. NIDUS-facilitators' session notes will be reviewed to further understand how the values, approaches and strategies were applied for specific dyads.
- Quantitative Trial data. Demographic, and baseline and 12-month follow-up main trial secondary measure data (facilitator GAS scores at 12-months, functional independence by Disability Assessment for Dementia scale, fidelity checklist data, quality of life for PLWD by DEMQol or proxy and carer by CarerQoL, Neuropsychiatric symptoms by Neuropsychiatric Inventory, Family carer anxiety and depression by Hospital Anxiety and Depression Scale, Apathy of PLWD by The Brief Dimensional Apathy Scale, and services used by Client Services Receipt Inventory) related to sampled dyads will be extracted from the trial database and used to describe the dyads included in the qualitative interviews. These data will not be statistically analysed.
- Researcher's reflexive field notes will be used to provide in-depth personal perspectives at the level of the dyad.

For peer review only - http://bmjopen.bmj.com/site/about/guidelines.xhtml

For trial-population level data we will collect the following from all intervention-arm participants (n=199):

- Family carers will be invited to complete an *'acceptability'* questionnaire (Appendix F) at 12-month follow-up, in which they will rate the extent to which their experiences of the intervention aligned with the core theoretical principles of the emerging theory of change.
- We will record trial data for dose (number of sessions), reach (sites and participant location), attrition (number of participants withdrawn) with measures summarised using appropriate tables and graphs.

We will also collect data for trial fidelity (adherence to manualised modules across a sample of 20% of intervention-arm dyads), and withdrawal data where possible from dyads who withdraw. Those who withdraw will either be sent a questionnaire with open questions (Appendix G) or invited to interview to capture/discuss the reasons for withdrawal (approx. 30 minutes). Observations (where available) for their sessions can be carried out to identify factors against the observation checklist (appendix E). If the dyad is unable to complete the withdrawal questionnaire, their facilitator will be asked to provide information regarding the reasons for withdrawal.

2.5 Data analysis.

Dyadic-level qualitative analysis. The qualitative dyadic interviews, facilitator interviews, qualitative observational data, and relevant facilitator field notes will be iteratively thematically analysed based on Braun and Clarke[15,16] six-phases of thematic analysis, to identify and analyse repeated patterns of meaning. Reflexive field notes will be triangulated with the findings to add depth and further insight.

Dyadic-level quantitative analysis. For observational data inter-rater reliability will be evaluated using the percentage of agreement and the kappa statistic. The quantitative observational checklist ratings for purposively sampled dyads will be used descriptively through tabulating numbers with percentages in each Likert category (strongly agree through to strongly disagree) for each item.

Baseline and 12-month Secondary measure trial data and demographic characteristics will be extracted from the final trial database for the purposively sampled interviewed dyads and used descriptively with measures summarised using appropriate tables and graphs.

Trial Population-level qualitative analysis. Acceptability qualitative (free text) data will be analysed thematically. This data will be used to understand convergence or divergence against matched constructs from dyadic-level findings.

Qualitative withdrawal data will be analysed thematically to identify patterns and themes and to better understand the reasons for withdrawing.

Trial Population-level quantitative analysis. Acceptability questionnaire ratings will be reported using descriptive statistics through tabulating numbers with percentages in each Likert category (strongly agree through to strongly disagree) for each item.

We will use summary statistics and graphs to describe participant locations (reach), number of sessions received (dose), and fidelity of delivery to manualised modules. We will report number (%) who withdraw from the intervention/study (attrition rate) and summarise characteristics of those who withdrew against those who did not. These data will be used to evaluate session numbers, geographical distribution and who withdraws.

In a subsequent quantitative study, after main study effectiveness analyses are complete, we will explore whether the number of sessions attended and acceptability scores are associated with intervention effectiveness as defined by 12-month carer-rated GAS scores; as well as exploring how these may differ between carers by sociodemographic characteristics, to understand who NIDUS-

For peer review only - http://bmjopen.bmj.com/site/about/guidelines.xhtml

For peer review only

Family works for. Analyses will involve fitting multiple regression models including adjustments for confounding factors.

2.6 Qualitative and quantitative integration.

A two-phased integration approach will be used to merge and interpret the findings. Phase one will integrate data at a dyadic-level and phase two will integrate dyadic-level findings with trial population-level data.

Dyadic-level data integration. A mixed-methods matrix will be used to triangulate all dyadic-level qualitative findings (dyad and facilitator interviews, observational data, and field notes) and quantitative data (secondary trial measures, observation checklist data), allowing the data to be openly, actively, and interactively considered in the context of each other[13] and 'talk to each other'.[12]. These data will be integrated to provide 'case-studies' (approx. N=30). Data will be summarised and displayed in a mixed-method matrix based on the meta-matrix.[17] This will enable analysis between types of data for a single dyad, and identification of patterns across all dyads. The matrix will be used to draw inferences and interpretations of how elements of the intervention interact at the level of the dyad through case-studies and sub-groups of high and low GAS score. This will help to explore factors that affect dyads' experiences of, and any benefit from, the intervention helping to gain understanding of how NIDUS-Family influences change in goal attainment at a dyadic-level. Generation of themes and patterns will be used to test, develop, and refine the emerging theory that the values and approaches are important, in combination with the strategies, in the success of the intervention for dyads.

Trial Population-level data integration. A joint display[18] will be used to integrate the findings from the dyadic-level matrix with the trial population quantitative and qualitative acceptability questionnaire outcomes and trial data for dose, reach, fidelity, and attrition data. This will be used to draw inferences and interpret how NIDUS-Family works at a 'population' level. This approach will enable mapping of dyadic-level data to trial population data to identify the essential mechanisms of impact, implementation and contextual factors that influence change, in turn, refining and consolidating the emerging theoretical model of change for dyadic goal attainment and the NIDUS-Family logic model.

3. **Ethics and dissemination**

The NIDUS-Family trial has been registered on the clinical trials register at http://www.isrctn.com/ISRCTN11425138. NIDUS-Family ethics, which cover this evaluation have been approved – Study Title: Clinical and cost-effectiveness of a New psychological intervention to support Independence in Dementia (NIDUS) for family carers and people living with dementia in their own homes: A randomised controlled trial REC reference: 19/LO/1667, IRAS project ID: 271363.

The evaluation findings will be disseminated through publications and conferences. They will also inform recommendations for the NIDUS-Family implementation study and strategy.

4. Discussion

This protocol follows the MRC guidance for process evaluations and outlines the rationale, design and methods for the process evaluation of the NIDUS-Family intervention. The focus of this evaluation is to identify the mechanisms of impact, implementation and contextual factors that influence goal attainment, and how these can help people with dementia live at home for longer. This evaluation is theory-driven and will test, develop, and refine the NIDUS-Family theory for goal attainment. Findings will be written up as recommendations that will feed into the NIDUS-Family implementation study.

Study Strengths. Following the MRC Guidance for complex interventions provides a framework for planning, designing, conducting, analysing, and reporting a process evaluation. This framework

For peer review only - http://bmjopen.bmj.com/site/about/guidelines.xhtml

provides a standardised approach to process evaluation enabling transparency across the relatively novel field of complex intervention process evaluation.

A convergent mixed-method design will combine qualitative and quantitative data iteratively to increase understanding of outcomes and improve the NIDUS-Family intervention for roll-out.[9,11] At interpretation level, the two-phase approach initially uses a mixed-method matrix to triangulate qualitative and quantitative data at the dyadic level, then a joint display to integrate dyadic-level findings with trial population data. Each level will draw out new insights and interpretations leading to a deeper understanding of how NIDUS-Family influences change through goal attainment.[19] The mixed-methods design will allow for the emerging theory to be tested, refined, and developed to elicit the key mechanisms of impact, implementation and contextual factors that influence goal attainment through NIDUS-Family. These findings will be used to inform the implementation study rolling out NIDUS-Family into practice to maximise its impact in the 'real world'.

The evaluator is independent from the trial, albeit funded by the NIDUS Programme. To enable understanding and awareness of the trial the evaluator has access to the NIDUS programme team, enabling effective communication and responsiveness to process changes. The evaluation outcomes will not feed into the ongoing trial, they will however inform the post-trial implementation study.

As the NIDUS-Family trial is implemented across various geographical locations, with emphasis on recruiting a diverse population, this reduces the presence of cohort effect.

The evaluator will keep a reflexive journal throughout to capture methodological and theoretical decisions and, transparency and reflection on data collection, analysis, and integration.

Study limitations. There are some limitations to this protocol, firstly, data will only be collected at 12-month follow-up, for some dyads there may be a lag between finishing the intervention and evaluation.

Secondly, as the NIDUS-Family intervention is a complex model, this process evaluation will evaluate a small sample of participants. As there are many contextual factors (dementia severity, covid, local resources, dyadic relationships) the findings are taken at a specific point in time and account for the contexts relating to that specific dyad so may not be generalisable to difference contexts. The evaluation will reflect and represent the geographical and cultural diversity of the NIDUS-Family trial, this may not be fully reflective of the underlying population.

Finally, it is important to note that even though the facilitators' data will be anonymised, the facilitators are employed by the trial and are involved in trial data collection.

Author's Contribution: Protocol written by Danielle Wyman and it was critically edited for intellectual content by Professor Laurie Butler (ARU), Professor Claudia Cooper (UCL), Professor Peter Bright (ARU), Dr Sarah Morgan-Trimmer(Exeter) and Dr Julie Barber (UCL).

Funding Statement: This work was carried out within the UCL Alzheimer's Society Centre of Excellence for Independence at home, NIDUS (New Interventions in Dementia Study) programme (Alzheimer's Society Centre of Excellence grant 330).

Competing Interests Statement: The NIDUS-Family trial is funded by the Alzheimer's Society Centre of Excellence grant 330, which is led by CC, with LB as a co-investigator. DW is a PhD student funded by the grant.

For peer review only - http://bmjopen.bmj.com/site/about/guidelines.xhtml

References

1 Alzheimer's Society. Alzheimer's Society's view on demography. April 29 2021 . Date accessed November 19, 2020 URLhttps://www.alzheimers.org.uk/about-us/policy-and-influencing/what-we-think/demography

2 Wittenberg R, Hu B, Barraza-Araiza L et al. Projections of older people with dementia and costs of dementia care in the United Kingdom, 2019–2040. 2019. CPEC Working Paper 5. Accessed April 29 2021, URL https://www.alzheimers.org.uk/sites/default/files/2019-11/cpec_report_november_2019.pdf

3 Prince M, Knapp M, Guerchet M, et al. (2014). Dementia UK: Update. Date accessed November 19, 2020 from https://www.alzheimers.org.uk/sites/default/files/migrate/downloads/dementia_uk_update.pdf

4 Lord K, Livingston G, Robertson S, et al. (2016). How people with dementia and their families decide about moving to a care home and support their needs: development of a decision aid, a qualitative study. *BMC Geriatric* 2016;16:1

5 England PH. Dementia: applying All our Health. London; 2018. Accessed November 19, 2020 URL https://www.gov.uk/government/publications/dementia-applying-all-our-health/dementia-applying-all-our-health

6 Lord K, Beresford-Dent J, Rapaport P, et al. Developing the New Interventions for independence in Dementia Study (NIDUS) theoretical model for supporting people to live well with dementia at home for longer: a systematic review of theoretical models and Randomised Controlled Trial evidence. *Social Psychiatry and Psychiatric Epidemiology;* 2020;55:1-14

7 Scott I, Cooper C, Leverton M, et al. Effects of nonpharmacological interventions on functioning of people living with dementia at home: A systematic review of randomised controlled trials. *International Journal of Geriatric Psychiatry* 2019;34:1386-1402

8 Rapaport P, Burton A, Leverton, M et al. "I just keep thinking that I don't want to rely on people." a qualitative study of how people living with dementia achieve and maintain independence at home: stakeholder perspectives. *BMC Geriatrics* 2020;20:5

9 Moore G F, Audrey S, Barker M et al. Process evaluation of complex interventions: Medical Research Council guidance. *BMJ* 2015;350:1258

10 Tashakkori A, Teddlie C. Sage Handbook of Mixed Methods in Social and Behavioural Research. London: Sage 2010: 725-748

11 Creswell J W, Creswell J D. Research Design: Qualitative, Quantitative, and Mixed Methods Approaches. 5th Edition London: Sage 2018.

12 Moseholm E, Fetters M D. Conceptual models to guide integration during analysis in convergent mixed methods studies. *Sage Journals* 2017;10:2

13 Alwashmi M F, Hawboldt J, Davis E et al. The Iterative Convergent Design for Mobile Health Usability Testing: Mixed Methods Approach. *JMIR mHealth and uHealth* 2019;7:4

14 Nelson J. Using conceptual depth criteria: addressing the challenge of reaching saturation in qualitative research. *Qualitative Research* 2016;17:5

15 Braun V, Clarke V. Using thematic analysis in psychology. *Qualitative Research in Psychology* 2006;3:77-101

16 Braun V, Clarke V. *Successful qualitative research; a practical guide for beginners*. London: Sage 2013

17 Wendler C. Triangulation using a meta-matrix. *Methodological Issues in Nursing Research* 2001;35:521-5

18 Guetterman T C, Fetters M D, Creswell J W. (2015). Integrating Quantitative and Qualitative Results in Health Science Mixed Methods Research Through Joint Displays. *Annals of Family medicine* 2015;13:6

19 Fetters M D, Curry L A, Creswell J W. Achieving integration in mixed methods designs principles and practices. *Health Services Research,* 2013;*48*:2134-2156

For peer review only - http://bmjopen.bmj.com/site/about/guidelines.xhtml

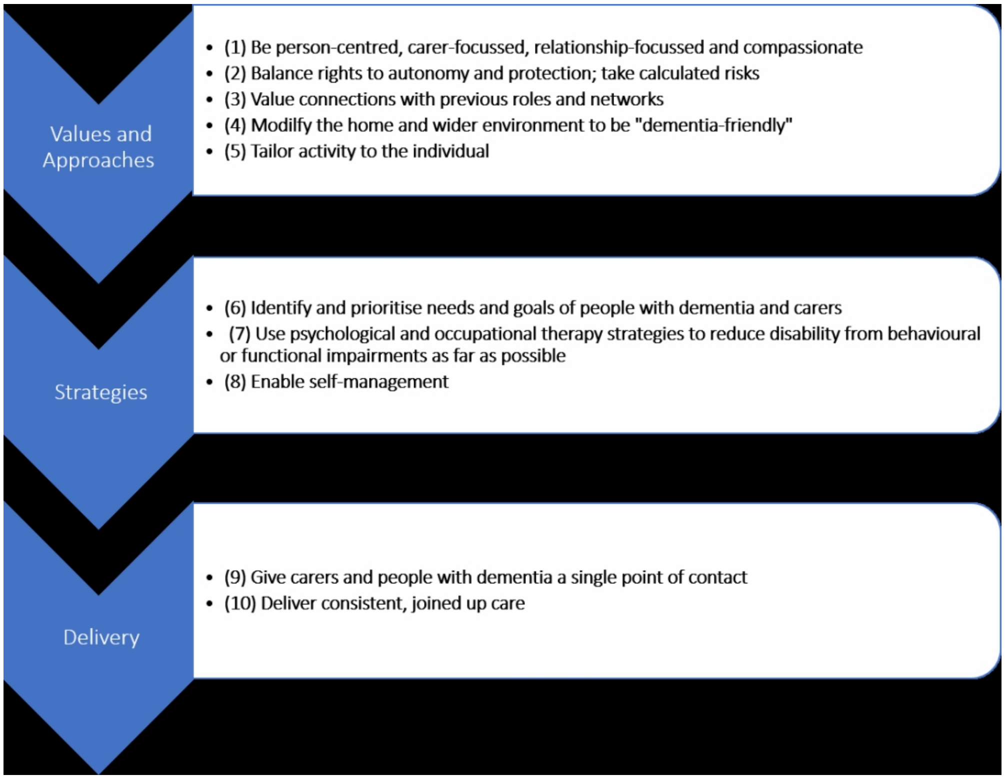

Figure 1. NIDUS Theoretical Model of Independence at Home

208x160mm (150 x 150 DPI)

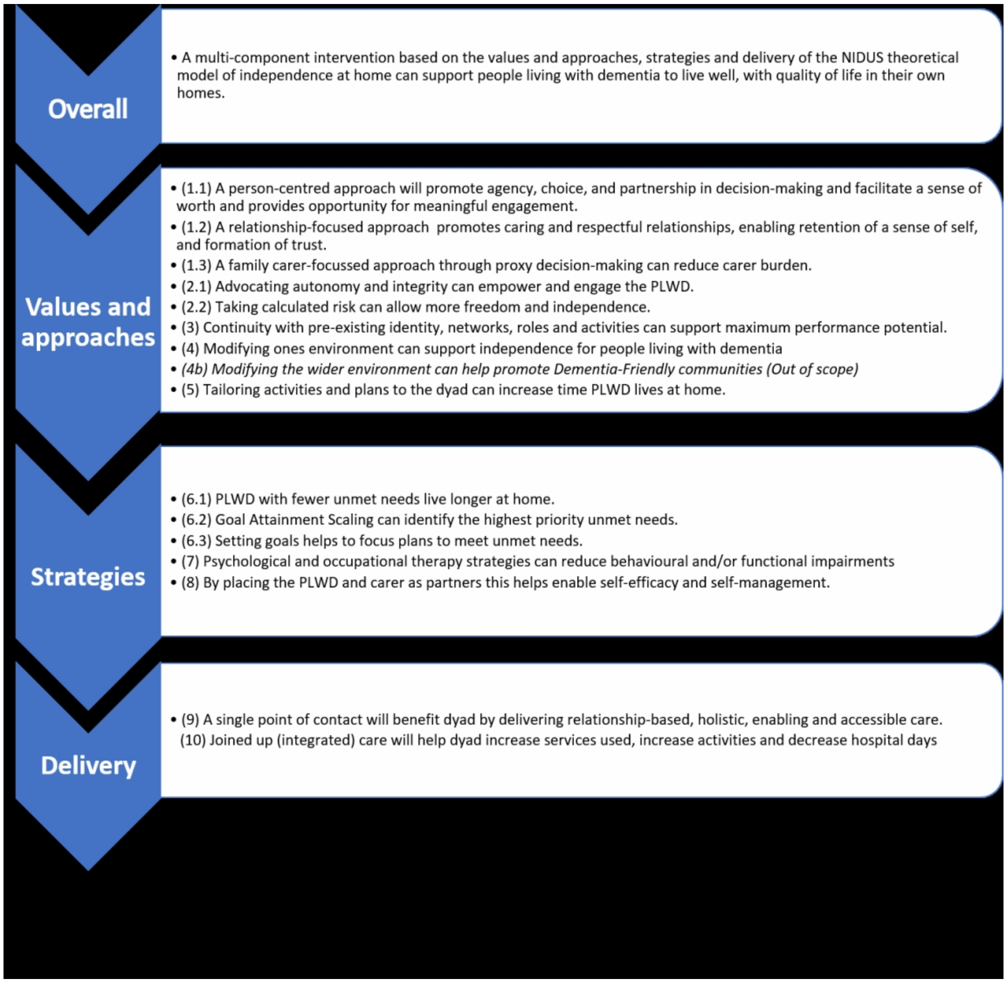

Figure 2.
Hypothesised NIDUS-Family Causal Assumptions

Note. Derived from NIDUS Theoretical Model of Independence at Home (Figure 1)

239x233mm (150 x 150 DPI)

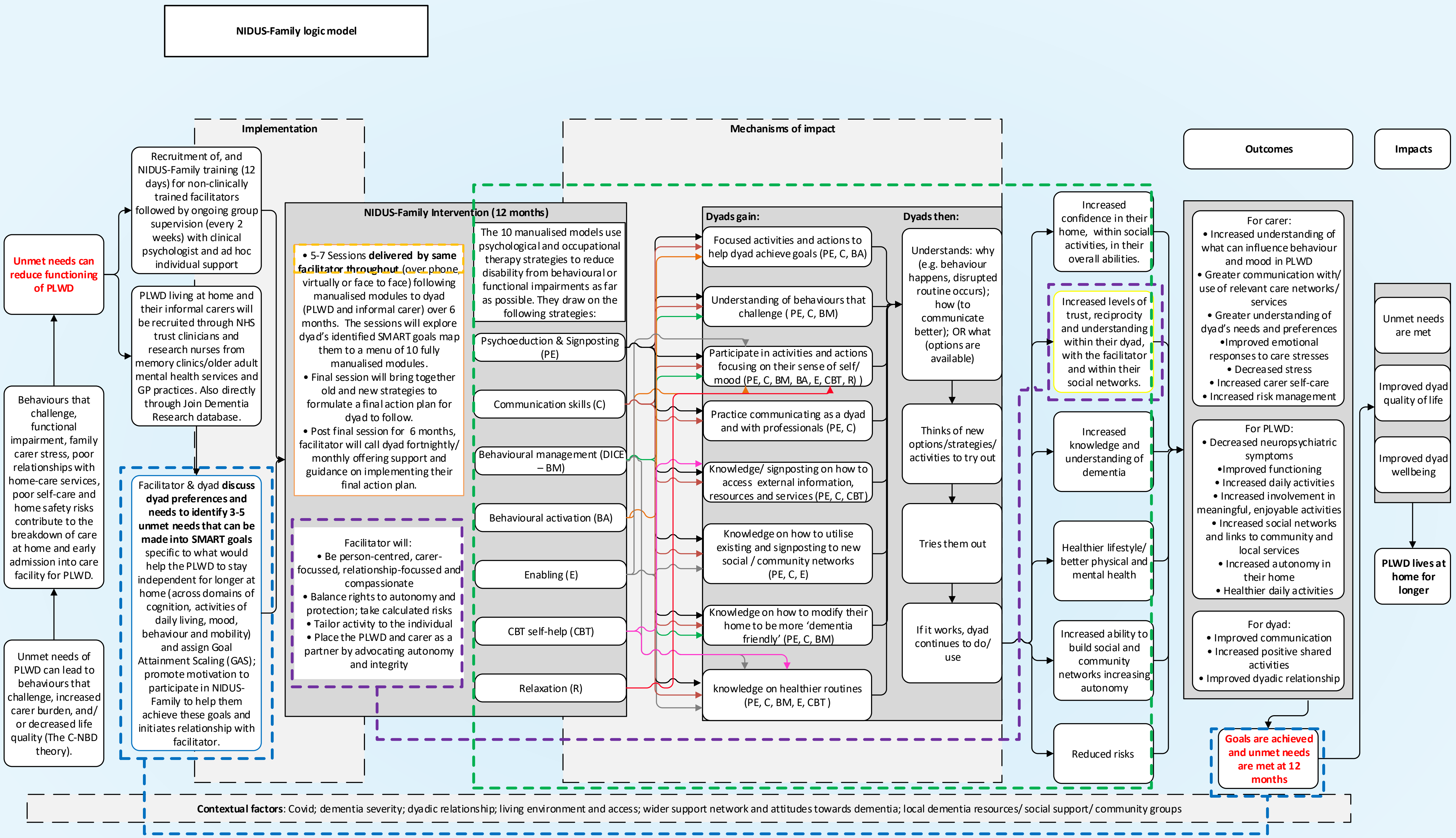
NIDUS-Family logic model
Implementation
Mechanisms of impact
Outcomes
Impacts
Unmet needs can reduce functioning of PLWD
Behaviours that challenge, functional impairment, family carer stress, poor self-care and relationships with home-care services, poor self-care and home safety risks contribute to the breakdown of care at home and early admission into care facility for PLWD.
Unmet needs of PLWD can lead to behaviours that challenge, increased carer burden, and/or decreased life quality (The C-NBD theory).
Recruitment of, and NIDUS-Family training (12 days) for non-clinically trained facilitators followed by ongoing group supervision (every 2 weeks) with clinical psychologist and ad hoc individual support
PLWD living at home and their informal carers will be recruited through NHS trust clinicians and research nurses from memory clinics/older adult mental health services and GP practices. Also directly through Join Dementia Research database.
Facilitator & dyad discuss dyad preferences and needs to identify 3-5 unmet needs that can be made into SMART goals specific to what would help the PLWD to stay independent for longer at home (across domains of cognition, activities of daily living, mood, behaviour and mobility) and assign Goal Attainment Scaling (GAS); promote motivation to participate in NIDUS-Family to help them achieve these goals and initiates relationship with facilitator.
NIDUS-Family Intervention (12 months)
• 5-7 Sessions delivered by same facilitator throughout (over phone, virtually or face to face) following manualised modules to dyad (PLWD and informal carer) over 6 months. The sessions will explore dyad's identified SMART goals map them to a menu of 10 fully manualised modules.
• Final session will bring together old and new strategies to formulate a final action plan for dyad to follow.
• Post final session for 6 months, facilitator will call dyad fortnightly/ monthly offering support and guidance on implementing their final action plan.
Facilitator will:
• Be person-centred, carer-focussed, relationship-focussed and compassionate
• Balance rights to autonomy and protection; take calculated risks
• Tailor activity to the individual
• Place the PLWD and carer as a partner by advocating autonomy and integrity
The 10 manualised models use psychological and occupational therapy strategies to reduce disability from behavioural or functional impairments as far as possible. They draw on the following strategies:
Psychoeduction & Signposting (PE)
Communication skills (C)
Behavioural management (DICE – BM)
Behavioural activation (BA)
Enabling (E)
CBT self-help (CBT)
Relaxation (R)
Dyads gain:
Focused activities and actions to help dyad achieve goals (PE, C, BA)
Understanding of behaviours that challenge (PE, C, BM)
Participate in activities and actions focusing on their sense of self/ mood (PE, C, BM, BA, E, CBT, R))
Practice communicating as a dyad and with professionals (PE, C)
Knowledge/ signposting on how to access external information, resources and services (PE, C, CBT)
Knowledge on how to utilise existing and signposting to new social / community networks (PE, C, E)
Knowledge on how to modify their home to be more 'dementia friendly' (PE, C, BM)
knowledge on healthier routines (PE, C, BM, E, CBT)
Dyads then:
Understands: why (e.g. behaviour happens, disrupted routine occurs); how (to communicate better); OR what (options are available)
Thinks of new options/strategies/ activities to try out
Tries them out
If it works, dyad continues to do/ use
Increased confidence in their home, within social activities, in their overall abilities.
Increased levels of trust, reciprocity and understanding within their dyad, with the facilitator and within their social networks.
Increased knowledge and understanding of dementia
Healthier lifestyle/ better physical and mental health
Increased ability to build social and community networks increasing autonomy
Reduced risks
For carer:
• Increased understanding of what can influence behaviour and mood in PLWD
• Greater communication with/ use of relevant care networks/ services
• Greater understanding of dyad's needs and preferences
• Improved emotional responses to care stresses
• Decreased stress
• Increased carer self-care
• Increased risk management
For PLWD:
• Decreased neuropsychiatric symptoms
• Improved functioning
• Increased daily activities
• Increased involvement in meaningful, enjoyable activities
• Increased social networks and links to community and local services
• Increased autonomy in their home
• Healthier daily activities
For dyad:
• Improved communication
• Increased positive shared activities
• Improved dyadic relationship
Goals are achieved and unmet needs are met at 12 months
Unmet needs are met
Improved dyad quality of life
Improved dyad wellbeing
PLWD lives at home for longer
Contextual factors: Covid; dementia severity; dyadic relationship; living environment and access; wider support network and attitudes towards dementia; local dementia resources/ social support/ community groups

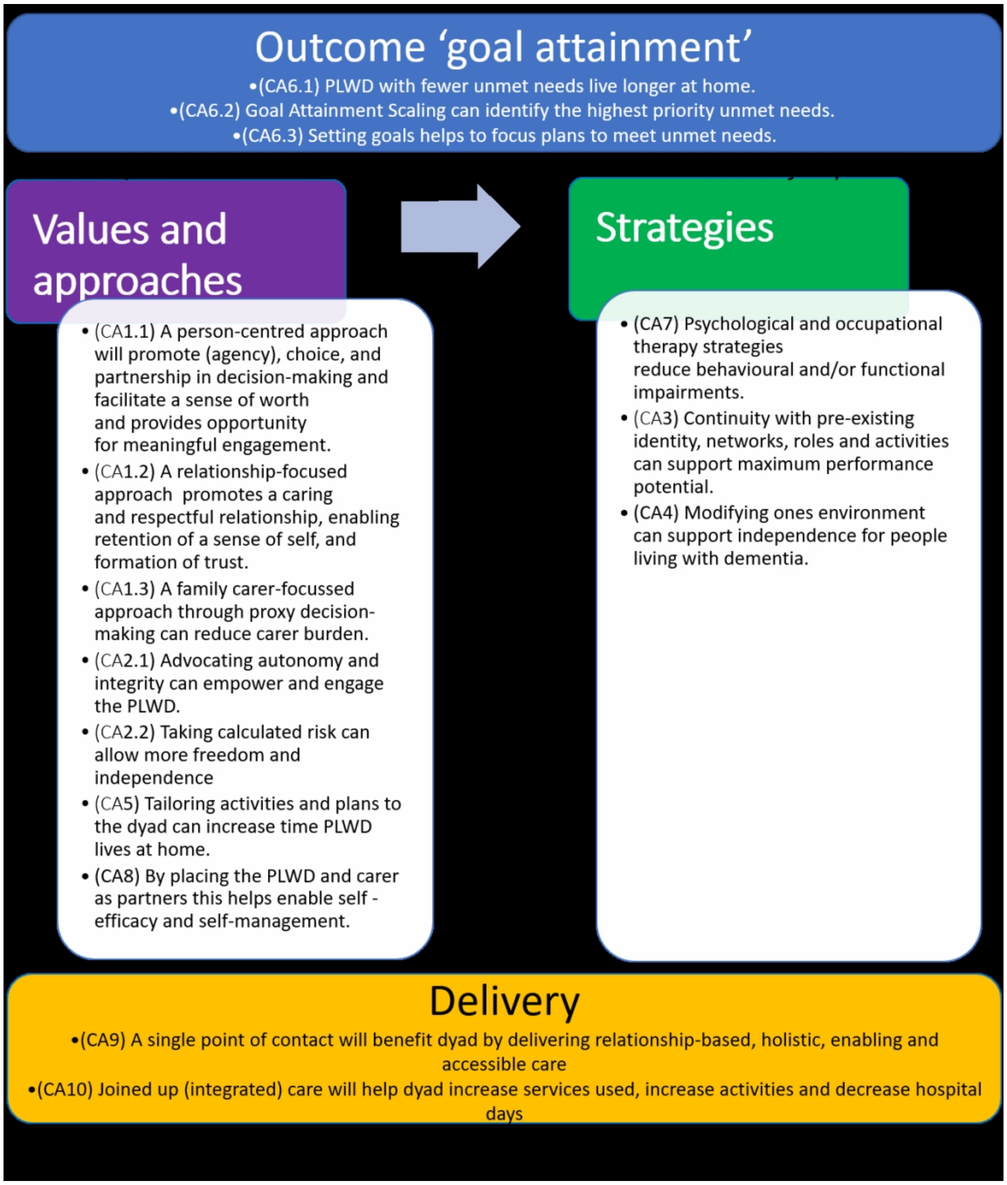

Figure 4.
Emerging Theoretical Model of Change for Attainment of Dyadic Goals through NIDUS-Family Intervention (with associated causal assumptions)

222x261mm (150 x 150 DPI)

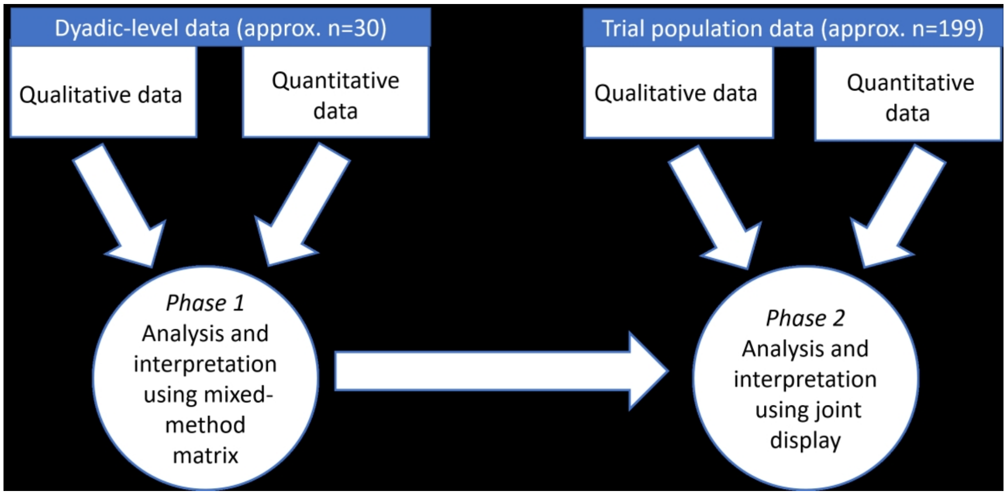

Figure 5.Mixed-Methods Convergent Design

388x189mm (150 x 150 DPI)

Appendix A

Alignment of planning, design and analysis of the NIDUS-Family process evaluation with MRC guidance.

Phase	MRC guideline recommendations (Moore et al, 2015)	Consideration of the recommendations for NIDUS-Family process evaluation
**Planning**	Define parameters of relationships of evaluators with intervention developers or implementers, balancing needs for good working relationships and independence; and how evaluators will inform implementation.	• Process evaluation led by a separate University.<br>• Evaluator is associate staff member at the trial University.<br>• NIDUS facilitators are employed through the intervention.<br>• Findings will inform the post-trial implementation strategy. They will not feed into the ongoing trial.
	Ensure the research team has the correct expertise, including, qualitative and quantitative research methods, and inter-disciplinary theoretical expertise.	Multi-disciplinary team includes expertise in psychology (ageing and behavioural change), old age psychiatry and dementia, neuropsychology, health service process evaluations, qualitative, quantitative, and mixed methods.
	Process and outcome evaluation team's degree of separation or integration:<br>• Oversight by a principal investigator.<br>• Good communication systems.<br>• Integration plans for process and outcome data agreed from the outset.	• Principal investigator has oversight over the NIDUS-Trial and is a subsidiary supervisor for evaluation lead.<br>•Evaluation is independent to the NIDUS-Family trial, but with weekly communication.<br>• Integration of process and outcome data will feed into the implementation study and strategy, but not into the trial.
**Designing**	Describe the intervention and its causal assumptions.	• The NIDUS-Family theory and causal assumptions are represented in a logic model (Figure 3).<br>• Section 1.1 describes the intervention, and 1.4 describes the causal assumptions
	• Identify questions by considering the intervention.<br>• Agree scientific and policy priority questions by considering the evidence for intervention assumptions.<br>• Consult with the evaluation team and policy/practice stakeholders.<br>• Identify previous process evaluations of similar interventions.	• The logic model informed the evaluation research questions.<br>• The multi-disciplinary team, including PPI, were consulted on the logic model.<br>• Relevant process evaluations were identified through a systematic review (PROSPERO ID: CRD42020221337).
	• Use quantitative methods to quantify key process variables and allow testing of pre-hypothesised	• Quantitative and qualitative methods will build upon one another to test, refine, and develop the NIDUS-Family

	mechanisms of impact and contextual moderators.<br>• Use qualitative methods to capture emerging changes in implementation, experiences of the intervention and unanticipated or complex causal pathways, and to generate new theory.<br>• Balance collection of data on key process variables from all sites or participants, with detailed case studies of purposively selected samples.<br>• Consider data collection at multiple time points to capture changes to the intervention over time.	logic model and emerging theory model (Figure 4).<br>• Quantitative methods will capture population level data on acceptability, reach, dose, attrition and secondary trial measures (approx. n=199). Quantitative observation data (approx. n=30) will enable detailed dyadic case-studies<br>• Qualitative interviews with purposively sampled dyads using GAS ratings (approx. N=30) will capture dyads experiences of receiving the intervention for case-studies and theme generation.<br>• Quantitative and qualitative methods will be matched on construct.<br>• Purposive sampling will recruit a sample representative of the trial population.<br>• Participants who withdraw will complete a questionnaire or an interview.<br>• Data collection at post 12-month follow-up for dyads and throughout for facilitators.
**Analysis**	Provide descriptive quantitative information on fidelity, dose and reach.	Fidelity: Fidelity checklist ratings for 20% of intervention-arm participants<br>Dose: number of sessions<br>Reach: Sites and locations<br>Attrition: Rate of withdrawal
	modelling of variations between participants or sites for factors such as fidelity or reach.	Contextual factors related to demographic data will be factored into data analysis and integration.
	Integrate quantitative process data into outcomes datasets, examining whether effects differ by implementation or pre-specified contextual moderators, and test hypothesised mediators.	Secondary trial data, dyadic observation fidelity checklist data, and acceptability ratings will be integrated to understand factors relating to high and low goal attainment.
	Collect and analyse qualitative data iteratively so that themes that emerge in early interviews can be explored in later ones.	Qualitative data collection and analysis will be carried out iteratively as dyads finish their 12-month follow-up. Emerging themes from earlier interviews will be explored in later interviews.
	quantitative and qualitative analyses build upon one another, with qualitative data used to explain	A two-stage integration approach will be used to merge the findings, initially at the

For peer review only

	quantitative findings, and quantitative data used to test hypotheses generated by qualitative data.	level of the dyad, then at the population level.
	Initially analyse and report qualitative process data prior to knowing trial outcomes to avoid biased interpretation.	Qualitative data will be collected and analysed before trial outcomes are known.
	Report whether process data are being used to generate hypotheses (analysis blind to trial outcomes), or for post-hoc explanation (analysis after trial outcomes are known).	Process data will be used to generate hypotheses, analysis will be blind to primary trial outcomes. Secondary outcomes will be analysed.

Note. Adapted from Moore et al (2015, p12)

For peer review only

Appendix B

Matching Quantitative and Qualitative Constructs Examples

**Construct**	**Associated causal assumptions**	**Quantitative questions**<br>*Method: Acceptability questionnaire and observation data collected through listening to video/audio recordings of dyads session*<br><br>*Variable: rate 1 Strongly disagree – 5 strongly agree)*		**Qualitative questions**<br>*Method: Qualitative semi-structured interviews*	
		Acceptability questionnaire<br><br>(Appendix F)	Observation checklist (Appendix E)	Dyad (Appendix C)	Facilitator (Appendix D)
Values and approaches	CA1.1		The facilitator promoted choice [for PLWD/ for Carer]	Do you feel you were able to contribute to the sessions?	Do you feel you promoted the dyad to have choice?
	CA1.1, 1.2, 8	[myself/ the person I care for] had a good relationship with my facilitator	Discussions were respectful/ sup portive [for PLWD/ for Carer] *(allowin g others to speak, actively listening, supporting their opinions, working as partners, discussing differing opinions calmly)*	• Do you feel you were respected?<br>•Do you feel you built up a level of trust with your facilitator?<br>• Do you feel the relationship was mutual and reciprocal?	• Do you feel you built a sense of trust with the dyad?<br>• Do you feel you actively listened?<br>• Do you feel there was mutual respect between you and the dyad?
	CA8		[PLWD/ Carer] had opportunities to ask questions.	How were your discussions in the sessions?	Who was involved in the discussion?
	CA1.3, 8	[I/ the person I care for] contributed to decision maki ng.	[PLWD/ Carer] contributed to decision ma king.	Did you feel involved in the decision-making?	Who was involved in decision-making?

For peer review only

	CA1.1	[I/ the person I care for] had opportunities for meaningful e ngagement.	[PLWD/ Carer] had opportunities for meaningful engagement *(a ble to actively participate, actively contribute ideas, skills or abilities)*	Do you feel you were able to actively participate?	Do you feel the dyad had opportunities for meaningful engagement?
	CA2.1		[facilitator/Car er] showed compassion *(di d they take time to bond, act with kindness, be encouraging, be polite)*	How did the facilitator make you feel?	How would you describe your persona in the sessions?
	CA2.2		[facilitator/PL WD/ Carer] explored risks [for PLWD/ for carer]	Did you discuss any possible risks?	Tell me about a risk you discussed and how you managed this (when setting tasks/ goals)?
	CA5	Goals were tailored to the [PLWD/ family member] nee ds.	The facilitator tailored [PLWD/ Carer] needs/goals/pl ans/activities/t asks.	• Do you feel the goals set reflected your needs/issues at the time?<br>• What plans, activities, tasks did you put in place to work towards your goals?	Once the goals were set can you talk through how you developed [plans/ activities/ actions] for the dyad to work towards their goals…
	CA1.3, 8		[facilitator/PL WD/ Carer] agreed (acknowledged) next steps (actions to follow the session)	Were you clear on activities between sessions?	*Who took accountability for actions?*
	CA8		The [PLWD/carer] acknowledged/ took ownership	Who took charge of doing the	Who took accountability for actions?

			for the actions/tasks s et.	activities/plan s?	
Goals and Strategies	CA6.3		Goals were discussed .	Tell me about your goals...	How did the [PLWD/carer] interact in the sessions?
	CA6.3	The modules helped [me/ the person I care for] work towards my goals...	Modules were discussed in line/ linked with the dyad's goals.	How did module [X] fit with/ affect your goals?	Talk me through how the modules worked for the dyad...
	CA6.3		[PLWD/ carer] engaged with the module/s...	How did you find the modules?	How do you feel the dyad engaged with the modules?
	CA6.3		Clear objectives/ next steps were set [for PLWD/Carer]	Who took charge of doing the activities/plan s?	Who took accountability for actions?

For peer review only

Appendix C

Semi-structured interviews for dyads

Interview Approach

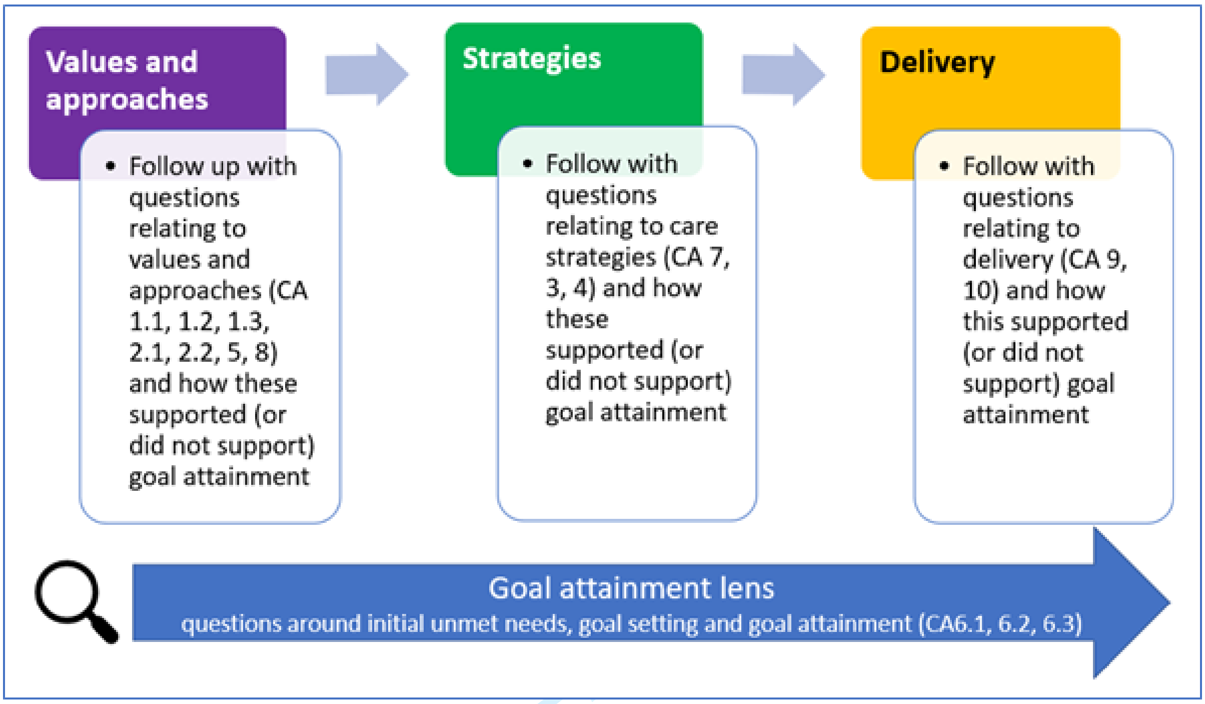

Semi-structured topic guide for dyads (PLWD and carer)

This will be used as a guide, a flexible approach will be adapted to capture any relevant information as well as unanticipated or unexpected pathways.

Introduction:

"Hello, I am XXXX and my role is XXXX. Thank you for your time today, I understand you have taken part in the NIDUS-Family intervention and I wanted to find out more about your experiences in receiving this. It would be interesting to explore your experiences of the NIDUS-Family trial, particularly in relation to your goals. There are no right or wrong answers, you are the experts. This interview will be audio-recorded and transcribed anonymously. Everything you say will be confidential and anonymised, any identifying information will be removed. You will be able to withdraw from this part of the study until the point of data analysis. Information from today will help us make sure any benefits of NIDUS-Family can be put into wider practice. Do you have any questions? If you are happy to continue, please can you give your consent (informed or by proxy) and I will start recording.".

Q1. Tell me about your experiences of NIDUS-Family… (where relevant ask PLWD first, then carer)

Prompts:

- How did you get involved with NIDUS-Family?
- What were your expectations of taking part in NIDUS-Family?

Q2. Tell me about the main things that have changed for you since finishing the NIDUS-Family intervention… *[ask PLWD and carer separately where possible]*

- How has that affected your day-to-day routine?
- How has that affected you?

Values and approaches

Q3. Tell me about your (PLWD and Carer) relationship?

- Overall:
  - *Day to day examples*
  - *Do you live together/ how often do you see each other?*
  - *Nature of visits?*
- NIDUS-Family specific
  - *How do you feel you worked together during the NIDUS sessions?*
  - *How were your discussions in the sessions?*
  - *Has your relationship changed since NIDUS-Family?*

Q4. Tell me about your relationship with the facilitator… (where relevant ask PLWD first, then carer)

  - *How did the facilitator make you feel?*
  - *Do you feel you were able to actively participate?*
  - *Do you feel you were able to contribute (ideas) to the sessions?*
  - *Did you feel involved in the decision-making?*
  - *Do you feel you built up a level of trust with your facilitator?*
  - *Do you feel the relationship was mutual and reciprocal?*
  - *Did you feel you partnered with the facilitator?*
  - *Do you feel you were respected?*
- Tell me how your relationship with your facilitator affected you attaining your goals?
- How did you feel about having the same facilitator throughout?

Q5. Tell me about your support network...

- informal – neighbours, friends, other family
- formal – services, resources
  - *Has this changed since starting/ finishing NIDUS-Family?*
  - *How did these (support/network/service use) changes affect your goals?*

Goal attainment

Q6. Tell me about your goals… (where relevant ask PLWD first, then carer)

Prompts:

- Do you feel the goals set reflected your needs/issues at the time?
  - *How did you discuss your needs?*
  - *Did you talk about your issues/needs?*
  - *Were they specific to you [PLWD and/or Carer]*
- Tell me more about how you decided on these goals?
  - *How were the goals set?*
  - *Who was involved in setting them?*
  - *How did that discussion go? Maybe we could use an example…*
- Do you feel your goals were achievable?
  - *[If not] Did you tell the facilitator you felt this at the time?*

For peer review only

- *Is this in hindsight?*
- Once you set your goals, tell me what you did next?
  - *How did you work towards your goals?*
  - *What plans, activities did you put in place?*
  - *Who came up with those plans?*
  - *Who took charge of doing the activites/plans?*
  - *Can you give me an example of something that helped?*
  - *Can you give me an example of something that may have got in your way?*
- Could you tell me why you scored your goals as (+2/ 0,-1,-2)?
- Knowing what you do now, is there anything you would have done differently?

Strategies

Q7. How did you find the modules (name modules *talk through one at a time*)?

- How did module [X] fit with your goals?
- How did module [X] affect your goals?
  - *What did you enjoy?*
  - *What did you not enjoy?*
- Were there any parts of the modules you liked?
- Were there any parts of the modules you didn't like?
- Is there anything you wish the modules had covered?

Q8. Before we finish, can I review the key points you mentioned about:

- Goal attainment
- Values and approaches
- Strategies
- Delivery

Q9. Is there anything else you would like to add?

Q10. Is there anything we haven't covered you feel is important?

Thank you for your time and for taking part today.

For peer review only

Appendix D

Semi-structured topic guide for NIDUS-Family facilitators

Interview approach

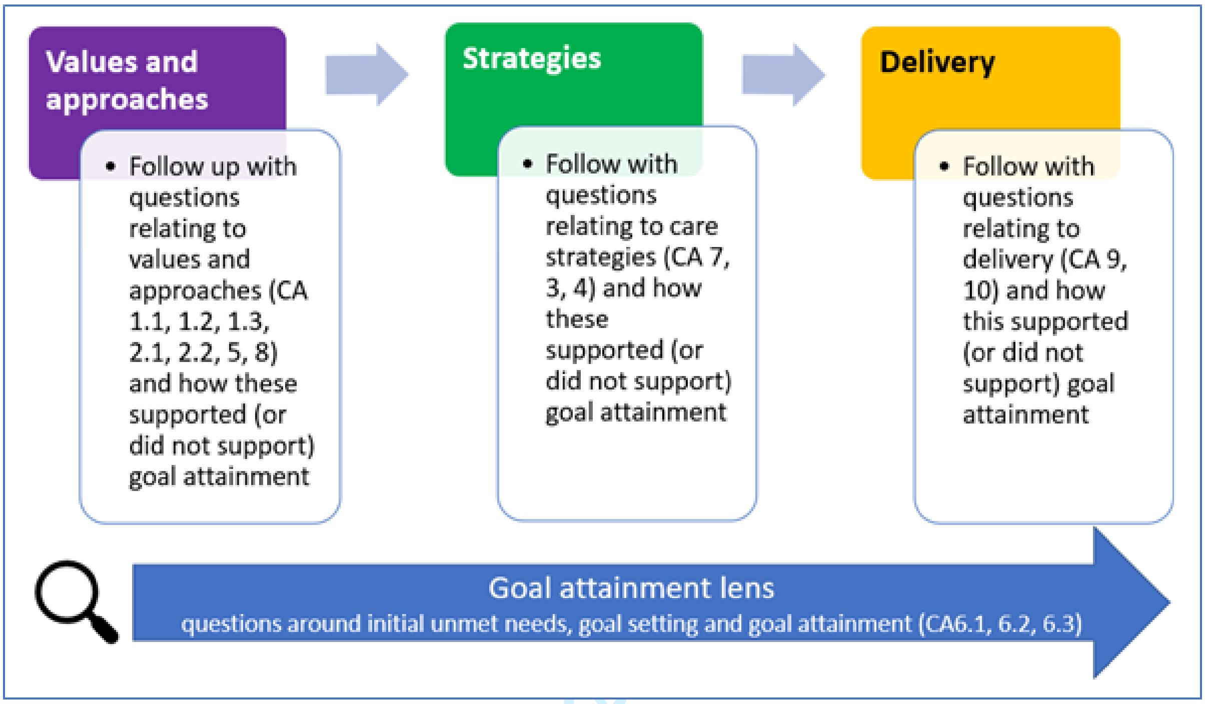

This will be used as a guide. A flexible approach will be adapted to capture any relevant information as well as unanticipated or unexpected pathways.

<u>Topic guide for facilitators</u>

Introduction:

"Hello, I am XXXX and my role is XXXX. Thank you for your time today, I wanted to find out more about your experiences in delivering the NIDUS-Family intervention. It would be interesting to explore things that you found went well and things that you feel could be improved to help us plan how this can be delivered in practice. There are no right or wrong answers, I'm interested in your experiences. This interview will be audio-recorded and transcribed, with your permission. Everything you say will be confidential and anonymised. Do you have any questions? If you are happy to continue, I will start recording.".

Q1. Can you tell me about the NIDUS-Family intervention?

- What are your experiences of the programme so far?
    - *Anything that was helpful?*
    - *Were there times when you felt uncomfortable delivering it?*
    - *Were there times the dyad felt uncomfortable receiving it?*

Q2. Can you tell me about your training?

- *How did you feel about delivering your first session?*
- *Can you give me an example…*

- Can you tell me what the top 'take homes' or 'key messages' you remember were from your training?
- Knowing what you do now, do you feel your training prepared you for your first session?
- Can you tell me about the support/ supervision you received through both training and when delivering?
  - *Anything that was helpful – can you give me an example?*
  - *What did you discuss with your supervision team – can you give me an example?*

Values and approaches

Q3. Can you tell me more about your relationship with dyad [XXX] across the sessions?

- *Do you feel you promoted the dyad to have choice (CA1.1)?*
- *Do you feel you promoted the dyad to have active agency? (CA1.2)*
- *Do you feel you built a sense of trust with the dyad? (CA1.2)*
- *Do you feel you actively listened?*
- *Do you feel your relationship provided mutuality and reciprocity? (CA1.2)*
- *Do you feel there was mutual respect between you and the dyad?*

- How do you feel your relationship affected the dyad attaining their goals?
- Do you feel the dyad had opportunities for meaningful engagement (able to actively participate, actively contribute ideas/ skills/abilities)?
- Can you tell me about the 'role' you played (parent-child relationship/ parent-parent relationship with dyad)?

Q4. Can you tell me about the dyads' relationship?

- *How did the dyad interact in the sessions?*
- *Who was involved in discussions?*
- *Who was involved in decision-making?*
- *Who took accountability for actions?*

- Expanding on the dyads' relationship – Can you give me an example of where the PLWD led on a suggestion or idea?

Goal attainment

Q5. Tell me about your experience of identifying issues/ needs with dyad XXXs…

- *How did you discuss this?*
- *Who led this conversation?*
- *Who identified the issues?*
- *How did dyads interact?*
- *Did dyads agree?*

- How were dyad [XXX] goals related to – PLWD/Carer or both?
- After you identified the dyad XXX '<u>needs</u>' tell me how you went about setting their goals…
  - *If it is easier, talk me through a specific example.*
  - *How did you link the need to their goal?*
  - *How was the dyad involved in the discussion?*

For peer review only

- *Was it PLWD and carer? Tell me more…*

Q6. Once the goals were set can you talk through how you developed [plans/ activities/ actions] for the dyad to work towards their goals…

- How were the dyad involved?

- Tell me about a risk you discussed and how you managed this (when setting tasks/ goals)?
- [If relevant] Dyad XXX rated themselves as [+2/ 0,-1,-2] could you tell me your views on why?
- [If relevant] The outcome assessor rated dyad [XXX] as [?] at 12-month follow up – do you agree? What would you have rated them?

Strategies

Q7. Talk me through how the modules worked for dyad [XXX]?

- What was your experience of delivering the manualised modules?
- How did you align dyad [XXX] goals to the modules?
- How do you feel the dyad [XXX] engaged with the modules?
- Can you give me an example where the modules helped motivate dyad [XXX]?
- Can you give me an example where the dyad did not understand or didn't 'click' with the module?
- *[if relevant]* Did you have discussions around adapting their home?
- *[if relevant]* Tell me about how adaptations to the dyads home affected them achieving their goals?

Delivery

Q8. What are your views on having one facilitator for each dyad?

To conclude

Q9. Before we finish, can I review the key points you mentioned about:

- **Goal attainment**
- **Values and approaches**
- **Strategies**

Q10. Is there anything else you would like to add?

Q11. Is there anything we have not covered you feel is important?

Thank you for your time and for taking part today.

For peer review only

Appendix E

Observation/ Fidelity Checklist

For fidelity checklist only complete italicised sections. Complete all sections for process evaluation observations.

Process evaluation factors	Please rate<br>1 Strongly disagree, 2 disagree, 3 neither agree nor disagree, 4 agree, 5 strongly agree	Examples – include descriptive text or quotations to demonstrate related observations<br>*(Free text)*
**Values and approaches (CA1.1, 1.2, 1.3, 2.1, 2.2, 5, 8)**		
[facilitator/PLWD/ Carer] paid attention (are they focused) in the session. [Fidelity checklist]	*For PLWD:*<br>*For Carer:*<br>*Facilitator:*	
**[facilitator/PLWD/ Carer] were engaged (actively contributed, followed the discussions) in the session.**	For PLWD:<br>For Carer:<br>Facilitator:	
**[facilitator/PLWD/ Carer] contributed to discussions.**	For PLWD:<br>For Carer:<br>Facilitator:	
**The facilitator promoted choice (CA1.1).**	For PLWD:<br>For Carer:<br>Overall:	
**Discussions were respectful (allowing others to speak, supporting their opinions, working as partners, discussing differing opinions calmly - CA1.1, 1.2 8).**	Between PLWD and carer:<br>Between PLWD and facilitator:<br>Between carer and facilitator:<br>Overall	
**[PLWD/ Carer] had opportunities to ask questions.**	PLWD:<br>Carer:	
**[PLWD/ Carer] contributed to decision making (CA1.3)**	For PLWD:<br>For Carer:<br>Overall:	
**[PLWD/ Carer] had opportunities for meaningful engagement (able to actively participate, actively contribute ideas, skills or abilities) for PLWD (CA1.1)**	For PLWD:<br>For Carer:	
**[facilitator/Carer] showed compassion (did they take time to bond, act with kindness, be encouraging, be polite - CA2.1)?**	Facilitator to PLWD:<br>Carer to PLWD:<br>Facilitator to carer:	
**[facilitator/PLWD/ Carer] explored risks (CA2.2).**	For PLWD:<br>For Carer:<br>For facilitator:<br>Overall:	

For peer review only

**the facilitator tailored [PLWD/ Carer] needs/goals/plans/activities/tasks (CA8).**	For PLWD:<br>For Carer:<br>Overall:	Needs:<br>Goals:<br>Plans:<br>Activities:<br>Tasks:
**[facilitator/PLWD/ Carer] agreed (acknowledged) next steps (actions to follow the session).**	PLWD:<br>Carer:<br>Facilitator:	
**Did the [PLWD/carer] acknowledge/ take ownership for the actions/tasks set?**	PLWD:<br>Carer:	
**Goals (CA6.1, 6.2, 6.3) and Strategies (CA7, 3, 4)**		
**Goals were discussed.**		Which goals?
**Modules were discussed in line/ linked with the dyad's goals.**		Which modules?
**Clear objectives/ next steps were set [for PLWD/Carer].**	For PLWD:<br>For carer	
**The facilitator kept the [PLWD/Carer] focused on the module/goal.**		
**Overall**		
**The group was relaxed.**		
**The facilitator kept the [PLWD/carer] engaged in the session.**	PLWD:<br>Carer	
**The facilitator kept the [PLWD/ carer] focused on the manual/goals.**	PLWD:<br>Carer:	
**The facilitator kept the session to time.**		

Any additional notes on the relationship dynamics between the:

- **Facilitator and PLWD**
- **Facilitator and carer**
- **PLWD and carer (Free text)**

For peer review only

**Any additional notes on the session (Free text)**
Any additional notes on impact of COVID-19 on delivery (Free text) [Fidelity checklist]
Any additional notes for modifications to the intervention or facilitator training (Free text) [Fidelity checklist]

For peer review only - http://bmjopen.bmj.com/site/about/guidelines.xhtml

APPENDIX F

Acceptability Questionnaire for Family Carers

Family carer ID ------------------------- Person living with dementia ID: --------------------------

GAS scores: ---------------------------------

NIDUS-Family acceptability scale: Family carer (Please tick the box you feel is the most relevant answer related to the question, rating from strongly disagree to strongly agree)

Statement		Strongly disagree	Disagree	Neither agree nor disagree	Agree	Strongly agree
The intervention helped the person I care for.						
[I/ the person I care for] contributed to decision-making.	Myself (family carer)					
	The person I care for					
[I/ the person I care for] had opportunities for meaningful engagement *(able to actively participate, actively contribute ideas, skills or abilities)*	Myself (family carer)					
	The person I care for					
Goals were tailored to [my/ the person I care for] needs.	Myself (family carer)					
	The person I care for					
The modules helped [me/ the person I care for] work towards my goals.	Myself (family carer)					
	The person I care for					
[myself/ the person I care for] had a good relationship with my facilitator.	Myself (family carer)					
	The person I care for					
The intervention helped improve my relationship with the person I care for.						

What feedback do you have for us about your experiences of receiving the NIDUS-Family intervention?

....

.... ***[Please turn over for more space]***

For peer review only

For peer review only

Appendix G

Withdrawal Questionnaire

Family carer ID ------------------------

Person living with dementia ID: --------------------------

**<u>What was the reason(s) you withdrew?</u>**
**<u>Would you do anything differently?</u>**
**<u>What would you change?</u>**

For peer review only

**Any other comments**

For peer review only

BMJ Open

Process evaluation of the New Interventions for independence in Dementia Study (NIDUS) Family stream randomised controlled trial: Protocol

Journal:	*BMJ Open*
Manuscript ID	bmjopen-2021-054613.R1
Article Type:	Protocol
Date Submitted by the Author:	28-Mar-2022
Complete List of Authors:	Wyman, Danielle; Anglia Ruskin University - Cambridge Campus, Faculty of Science and Engineering<br>Butler, Laurie; Anglia Ruskin University - Cambridge Campus, Psychology<br>Cooper, Claudia; University College London, Psychiatry of Older Age<br>Bright, Peter; Anglia Ruskin University, Psychology<br>Morgan-Trimmer, Sarah; University of Exeter Medical School, Institute of Health Research<br>Barber, Julie; UCL, Statistical Science
**Primary Subject Heading**:	Geriatric medicine
Secondary Subject Heading:	Research methods
Keywords:	Dementia < NEUROLOGY, Old age psychiatry < PSYCHIATRY, Clinical trials < THERAPEUTICS

SCHOLARONE™
Manuscripts

TITLE PAGE

TITLE: Process evaluation of the New Interventions for independence in Dementia Study (NIDUS) Family stream randomised controlled trial: Protocol

Corresponding author

Full Name: Danielle Laura Wyman

Address: 8 Wren Close, Ringwood, BH24 3RF

Email: dlw136@pgr.aru.ac.uk

Co-authors

Professor Laurie Butler, Psychology, Anglia Ruskin University, Cambridge.

Professor Claudia Cooper, Psychiatry of Older Age, University College London, London.

Professor Peter Bright, Psychology, Anglia Ruskin University, Cambridge

Dr Sarah Morgan-Trimmer, Institute of Health Research, University of Exeter.

Dr Julie Barber, Statistical Science, University College London.

Total word count: 4,000

Keywords: Process evaluation, dementia, quality of life,

ABSTRACT:

Introduction: NIDUS-Family is an Alzheimer's Society funded new manualised, multimodal psychosocial intervention to support People Living with Dementia (PLWD) to achieve goals that they and their family carers set, towards living as independently and as well as possible at home for longer. This process evaluation will be embedded within the NIDUS-Family Randomised Controlled Trial intervention-arm (n=199), testing how the intervention influences change, as measured by goal attainment. The evaluation will test, refine, and develop the NIDUS-Family theoretical model, associated causal assumptions and logic model to identify key mechanisms of impact, implementation and contextual factors influencing the intervention's effectiveness. Findings will inform how the program is implemented in practice.

Methods and analysis: The process evaluation will be theory-driven and apply a convergent mixed-methods design. Dyads (PLWD and family carer) will be purposively sampled based on high or low Goal Attainment Scaling scores (trial primary outcome). Qualitative interviews with dyads (approx. n=30) and their respective facilitators post-trial will explore their experiences of receiving and delivering the intervention. Interviews will be iteratively thematically analysed. *Matching* observational quantitative data will be collected concurrently from video and/or audio recordings of NIDUS-Family dyad trial sessions. Further quantitative data will be collected through an acceptability questionnaire for all intervention-arm dyads (n=199). Mixed-method integration will use an interactive analysis strategy, considering qualitative and quantitative findings through mixed-method matrix for dyadic level 'case-studies', and a joint display for 'population' level analysis and interpretation.

Ethics and dissemination: Ethical approval was received from Camden & Kings Cross Research Ethics Committee (REC). Study reference: 19/LO/1667. IRAS project ID: 271363. This work is carried out within the UCL Alzheimer's Society Centre of Excellence (grant 300) for Independence at home, NIDUS (New Interventions in Dementia Study) programme.

For peer review only

Findings will be disseminated through publications and conferences, and as recommendations for the implementation study and strategy.

TRIAL REGISTRATION DETAILS: Information on the NIDUS-Family RCT is available on the clinical trials register http://www.isrctn.com/ISRCTN11425138

STRENGTHS AND LIMITATIONS OF THIS STUDY

• This evaluation will place people living with dementia and their family carer as experts to inform how NIDUS-Family is implemented in practice.

• This evaluation will use a convergent mixed-methods design grounded within a theory-informed logic model and will follow the Medical Research Council process evaluation guidelines.

• The researcher carrying out this process evaluation is independent from the trial, albeit funded by the NIDUS Programme.

• Data collection occurs post trial, so there may be a time-lag between dyad finishing the trial and data collection which may affect recall of experiences.

• Qualitative interviews will occur with approximately 15% of dyads from the intervention-arm, such that the results may not be generalisable across other dyads.

For peer review only

1 INTRODUCTION

Dementia is a syndrome affecting multiple aspects of a person's cognitive function.[1] Currently an estimated 885,000 people are living with dementia in the UK, and this number is predicted to increase to over 1.2 million by 2030.[2] Approximately two thirds of people with dementia are living in their own homes,[3] and wish to remain doing so as independently as possible.[4]

Occupational and psychosocial therapy-based interventions are recommended by the UK National Institute for Health and Care Excellence (NICE) to promote wellbeing and independence for all people with dementia.[5] Muti-component interventions have demonstrated positive outcomes on a range of measures for PLWD and their carers [6]. Due to the complex nature of dementia, outcomes that matter most to PLWD and their carers vary between individuals and over time[6].

Challenging or distressing behaviours—also known as neuropsychiatric symptoms (NPS)—associated with dementia can lead to family carer stress, poor relationships with home care services, poor self-care, home safety risks and increased health care costs and are common reasons why people living with dementia move to a care home.[4] A systematic review of the effectiveness of psychosocial interventions for managing NPS showed given the positive outcomes, individualised behavioural interventions are possibly efficacious in reducing NPS, and that perceived management of NPS may change, resulting in reduced carer distress, and overall cost of care.[6] The review also concluded the most promising interventions for managing NPS seem to be individually tailored behavioural interventions, although more evidence and further research is advised.[6] There is a need to establish an evidence base for interventions in improving personalised support, adaptations, independence, and quality of life of people living with dementia[7].

1.1 The New Interventions for Independence in Dementia Study (NIDUS): Family

The NIDUS-Family program is a new manualised, multimodal psychosocial intervention to support people living with dementia to live independently at home for as long, and as well as possible. The intervention focuses on behavioural change, and aims to promote living with quality of life, choice, autonomy, dignity and as independently as possible.[8] The trial's primary objectives are to evaluate the effect of NIDUS-Family and routine care, relative to routine care alone at 12-month follow up, on goal attainment as measured by family-carer rated Goal Attainment Scaling (GAS) scores, and its cost-effectiveness. Secondary outcomes will measure activities of daily living, quality of life, neuropsychiatric behaviours, apathy, anxiety and depression, and service receipt.[8]

The NIDUS-Family intervention is founded upon several theoretical principles (Figure 1).

Figure 1.

NIDUS Theoretical Model of Independence at Home

Note. Lord et al (2020)

The NIDUS-Family intervention will recruit 297 participants with a diagnosis of dementia, living in their own home and their regular family carer, who is in at least weekly (including remote) contact. Randomisation will be blocked and stratified by site using a 2:1 intervention: routine care allocation ratio. Consent processes, outcome assessments and intervention delivery will be conducted over 12-months in the participant's own home, at the offices of the recruiting facilitator or via telephone or video call, depending on individual participant preference and COVID-19 restrictions.

The participants randomised to the active intervention group (n=199) will receive between six-to-eight manualised sessions within the first six months. NIDUS-Family aims to support people with dementia and their family carers (a dyad) to address personalised goals aligned to living as well as

For peer review only

possible at home.[8] The manualised sessions will be tailored to each participant dyad depending on their preferences and needs and all are delivered by the same facilitator where possible, and audio recorded. The facilitators (graduate psychologists and social researchers with relevant experience but without formal clinical training) will be trained—with three days dedicated to research procedures and nine days to intervention delivery—on how to deliver the intervention and supervised throughout by a clinical psychologist.

Full details relating to the recruitment, design, and delivery can be found in the NIDUS Study protocol.[8]

The NIDUS-Family intervention can be defined as a complex intervention due to its multiple interacting components, including the personalised and tailored approach, the dyadic relationship, and the differing contexts such as living arrangements, within which the programme is implemented.

1.2 Process evaluation of the NIDUS-Family intervention

Process evaluations aim to provide a detailed understanding of an intervention to inform policy and/or implementation into practice. The Medical Research Council (MRC) guidance [9] recommends examining aspects of the intervention including *"implementation (the structures, resources and processes through which delivery is achieved, and the quantity and quality of what is delivered), mechanisms of impact (how the intervention activities, and participants' interactions with them, trigger change), and context (how external factors influence the delivery and functioning of interventions)"* [9, p10].

This process evaluation will use the MRC's systematic approach for planning, design, analysis, and reporting (Appendix A shows alignment to the guidance).[9]

1.3 Process evaluation aims and objectives

As recommended by the MRC guidelines, this process evaluation will apply a theory-driven approach to respond to the research question: how does the NIDUS-Family intervention influence goal attainment?

We will explore how the hypothesised causal chains interact, to test and generate theory about how the NIDUS-Family intervention influences change through:

1) Evaluating the mechanisms of impact, implementation, and contextual factors comprising the NIDUS-Family intervention, with a primary focus on factors relating to goal attainment.
2) Identifying which mechanisms, implementation and contextual factors are essential for influencing the effectiveness of the NIDUS-Family intervention.

1.4 Theoretical basis

The NIDUS theoretical model (Figure 1), and its underpinning theories [6] directly informed the development of the hypothesised NIDUS-Family intervention causal assumptions (Figure 2).

Figure 2.

Hypothesised NIDUS-Family Causal Assumptions

Note. Derived from NIDUS Theoretical Model of Independence at Home (Figure 1)

For peer review only

The NIDUS-Family logic model (Figure 3) in turn clarifies how the NIDUS-Family intervention is designed to realise its intended outcomes and overlays the related causal assumption (CA) pathways for goal attainment, values and approaches, strategies, and delivery.

Figure 3.

NIDUS-Family Logic Model

The logic model (Figure 3) represents how NIDUS-Family helps dyads to identify three-to-five unmet needs related to living for longer at home. These are turned into specific, measurable, attainable, relevant, and time-bound (SMART) goals. The personalised goals are then mapped onto the NIDUS-Family manualised modules. The dyad attends six to eight sessions with a facilitator to work through the modules, bringing together old and new strategies to formulate a final action plan for dyads to follow to help them attain their goals. The intended outcomes for dyads are to improve communication, increase positive shared activities and improve their overall dyadic relationship. By attaining their goals, their unmet needs will now be met, leading to improved quality of life, wellbeing and the PLWD living at home for longer.

The logic model overlays the hypothesised NIDUS-Family causal pathways (Figure 2), the blue pathway represents causal assumptions linked to goal attainment, the purple pathway to values and approaches, the green pathway to strategies, and the yellow pathway represents causal assumptions associated with delivery. Overlaying the causal assumptions onto the logic model details how NIDUS-Family works based on theory and highlights the key pathways that are intended to influence change and will form the focus of this process evaluation.

This process evaluation will evaluate how the pathways delineated in the logic model (Figure 3) work in practice. It will test the emerging theory of change for attainment of dyadic goals (Figure 4) which represents how the core theoretical principles and casual assumptions derived from the logic model influence behavioural change through goal attainment and posits "*NIDUS-Family supports dyads to attain their goals through applying values and approaches, and strategies, supported by delivery through a single point of contact, and consistent joined up care*." Focusing on dyads with high (2) and low (0 or below) 12-month carer-rated GAS scores will identify how the intervention works and enable theory development and refinement.

Figure 4.

Emerging Theoretical Model of Change for Attainment of Dyadic Goals through NIDUS-Family Intervention (with associated causal assumptions)

2. Methods

2.1 Design.

A pragmatic paradigm—creating shared meaning and joint action [10]—will underpin the methodology to understand how the NIDUS-Family intervention works. To test, explore, refine, and develop the emerging NIDUS-Family theory of change (Figure 4) for dyadic goal attainment, and associated hypothesised causal assumptions, a convergent mixed-methods design will be applied (Figure 5).[9, 11] This design will integrate qualitative and quantitative data— dyadic-level data will be triangulated using a mixed-method matrix to help identify trends and patterns, and population-level data will be integrated with the dyadic-level findings using a joint-display (see section 2.7)—to help gain a more complete understanding of how NIDUS-Family, if effective, influences behavioural,

For peer review only

lifestyle and environmental change to enable goal attainment. Qualitative and quantitative questions will be matched on the emerging theory of change constructs for values and approaches, strategies and delivery through a lens of goal attainment (Appendix B shows matched constructs).[12]

Figure 5.

Mixed-Methods Convergent Design

2.2 Patient and Public Involvement

NIDUS-Family intervention stakeholders (NIDUS clinical psychologists, statisticians, facilitators and the programme manager) and the Patient and Public Involvement (PPI) group (eight Alzheimer's Society Research Network Volunteers) were consulted in the development of the NIDUS-Family logic model. Consultation occurred in various stages via presentation to map out how the NIDUS-Trial intends to work based on the theory. Feedback was captured and inputted to create the logic model.

2.3 Sampling

For dyadic-level data, dyads will be purposively sampled using quantitative primary measure trial data for 12-month follow-up family-carer rated GAS scores. Dyads with high goal attainment (a score of +2), and dyads with low goal attainment (a score of 0 or below) scores will be invited to interview and their recorded trial sessions (minimum one where available) will be observed through watching/listening to recordings. Dyads' respective facilitators will be invited to interview. To ensure sufficient conceptual depth, the conceptual depth scale[13] based on range, complexity, subtlety, resonance, and validity, will be applied to guide sample size for the number of dyads to be interviewed (approx. 10% N=30). Facilitators will be invited to a second interview when sufficient conceptual depth for dyads is reached to capture any data for subsequently sampled dyads they facilitated. Sampling for high and low 12-month carer-rated GAS scores will help us to understand what influences high scoring dyads to attain their goals, and why low scoring dyads do not attain their goals. This will help us to explore and identify the causal factors which contribute to people benefiting from the intervention.

For trial population-level data, all carers from the intervention-arm will be sent/ invited to complete via telephone call, an acceptability questionnaire at 12-month follow up. Relevant trial data for dose, reach, and attrition will be collected for all intervention-arm dyads. Data will be extracted from the final locked trial database after completion and analysed descriptively. This will help us to understand how the NIDUS-Family intervention influences goal attainment at the trial population level. Fidelity checklists will be applied to a sample of 20% of the intervention-arms transcribed trial session audio/video recordings.

2.4 Consent

Trained NIDUS-Family facilitators will assess capacity to consent and obtain written informed consent from each family carer and PLWD prior to NIDUS-Family trial participation. Family carers of people who lack capacity to consent will be asked to complete a consultee declaration form on behalf of their relative with dementia.

Family carers and PLWD will be asked at their 12-month follow up if they consent to being contacted by the process evaluation researcher. Those that give consent will be contacted and invited to interview to talk about their experiences of receiving NIDUS-Family as part of this process evaluation study. Where the PLWD lacks capacity, interviews will take place with the carer only.

2.5 Data collection

For peer review only

For dyadic-level data collection (intervention-arm dyads sampled for high (+2) and low (0 or below) carer-rated GAS scores at 12-month follow-up) we will collect:

- Qualitative semi-structured Interviews. Purposively sampled dyads, and their facilitator will be invited to separate interviews. Dyad interviews and facilitator interviews will be audio recorded, anonymised with pseudonyms, transcribed verbatim and uploaded onto NVivo 12 to manage the data analysis process.
    - The dyad semi-structured qualitative interview (see Appendix C for topic guide) will explore their experiences of how the NIDUS-Family approaches and values, and strategies influenced them in attaining their goals for high GAS scores (2+), and why low GAS scores (0 or below) had no change or did not attain their goals.
    - The semi-structured, qualitative interview with facilitators (see Appendix D for topic guide) will explore key factors they feel influenced dyadic goal attainment for the dyad(s) selected to whom they delivered the intervention, as well as their overall experiences of facilitating NIDUS-Family. Any novel data relevant to subsequent sampled dyads will be explored with the respective facilitator in a second interview when sufficient conceptual depth for dyads has been captured.
- Observational data for purposively sampled dyads attending interview. The evaluator will listen to the dyads' recorded trial sessions (minimum one session per dyad where available). Qualitative (aligned to 'free-text' sections) and quantitative (aligned to Likert scale ratings) data relating to the emerging theory will be captured in an observation checklist (Appendix E). To ensure validity, a second researcher will independently complete the observation checklist for a minimum of 10% of observed sessions and these observations may be drawn upon in the facilitator and dyad interviews. NIDUS-facilitators' session notes will be reviewed to further understand how the values, approaches and strategies were applied for specific dyads.
- Quantitative Trial data. Demographic, and baseline and 12-month follow-up main trial secondary measure data (facilitator GAS scores at 12-months, functional independence by Disability Assessment for Dementia scale, fidelity checklist data, quality of life for PLWD by DEMQol or proxy and carer by CarerQoL, Neuropsychiatric symptoms by Neuropsychiatric Inventory, Family carer anxiety and depression by Hospital Anxiety and Depression Scale, Apathy of PLWD by The Brief Dimensional Apathy Scale, and services used by Client Services Receipt Inventory) related to sampled dyads will be extracted from the trial database and used to describe the dyads included in the qualitative interviews. These data will not be statistically analysed.
- Researcher's reflexive field notes will be used to provide in-depth personal perspectives at the level of the dyad.

For trial-population level data we will collect the following from all intervention-arm participants (n=199):

- Family carers will be invited to complete an *'acceptability'* questionnaire (Appendix F) at 12-month follow-up, in which they will rate the extent to which their experiences of the intervention aligned with the core theoretical principles of the emerging theory of change.
- We will record trial data for dose (number of sessions), reach (sites and participant location), attrition (number of participants withdrawn) with measures summarised using appropriate tables and graphs.

We will also collect data for trial fidelity (adherence to manualised modules across a sample of 20% of intervention-arm dyads), and withdrawal data where possible from dyads who withdraw. Those who withdraw will either be sent a questionnaire with open questions (Appendix G) or invited to interview to capture/discuss the reasons for withdrawal (approx. 30 minutes). Observations (where available) for their sessions can be carried out to identify factors against the observation checklist

For peer review only

(appendix E). If the dyad is unable to complete the withdrawal questionnaire, their facilitator will be asked to provide information regarding the reasons for withdrawal.

Data will be collected from August 2021 through to May 2023 by the process evaluation lead researcher, a postgraduate student who has extensive experience carrying out qualitative interviews with PLWD.

2.6 Data analysis.

Dyadic-level qualitative analysis. The qualitative dyadic interviews, facilitator interviews, qualitative observational data, and relevant facilitator field notes will be iteratively thematically analysed based on Braun and Clarke[14,15] six-phases of thematic analysis, to identify and analyse repeated patterns of meaning. Reflexive field notes will be triangulated with the findings to add depth and further insight.

Dyadic-level quantitative analysis. For observational data inter-rater reliability will be evaluated using the percentage of agreement and the kappa statistic. The quantitative observational checklist ratings for purposively sampled dyads will be used descriptively through tabulating numbers with percentages in each Likert category (strongly agree through to strongly disagree) for each item.

Baseline and 12-month Secondary measure trial data and demographic characteristics will be extracted from the final trial database for the purposively sampled interviewed dyads and used descriptively with measures summarised using appropriate tables and graphs.

Trial Population-level qualitative analysis. Acceptability qualitative (free text) data will be analysed thematically. This data will be used to understand convergence or divergence against matched constructs from dyadic-level findings.

Qualitative withdrawal data will be analysed thematically to identify patterns and themes and to better understand the reasons for withdrawing.

Trial Population-level quantitative analysis. Acceptability questionnaire ratings will be reported using descriptive statistics through tabulating numbers with percentages in each Likert category (strongly agree through to strongly disagree) for each item.

We will use summary statistics and graphs to describe participant locations (reach), number of sessions received (dose), and fidelity of delivery to manualised modules. We will report number (%) who withdraw from the intervention/study (attrition rate) and summarise characteristics of those who withdrew against those who did not. These data will be used to evaluate session numbers, geographical distribution and who withdraws.

In a future quantitative study, after main study effectiveness analyses are complete, we will explore whether the number of sessions attended and acceptability scores are associated with intervention effectiveness as defined by 12-month carer-rated GAS scores; as well as exploring how these may differ between carers by sociodemographic characteristics, to understand who NIDUS-Family works for. Analyses will involve fitting multiple regression models including adjustments for confounding factors.

2.7 Qualitative and quantitative integration.

A two-phased integration approach will be used to merge and interpret the findings. Phase one will integrate data at a dyadic-level and phase two will integrate dyadic-level findings with trial population-level data.

Dyadic-level data integration. A mixed-methods matrix will be used to triangulate all dyadic-level qualitative findings (dyad and facilitator interviews, observational data, and field notes) and quantitative data (secondary trial measures, observation checklist data), allowing the data to be

For peer review only

openly, actively, and interactively considered in the context of each other[16] and 'talk to each other'.[12]. These data will be integrated to provide 'case-studies' (approx. N=30). Data will be summarised and displayed in a mixed-method matrix based on the meta-matrix.[17] This will enable analysis between types of data for a single dyad, and identification of patterns across all dyads. The matrix will be used to draw inferences and interpretations of how elements of the intervention interact at the level of the dyad through case-studies and sub-groups of high and low GAS score. This will help to explore factors that affect dyads' experiences of, and any benefit from, the intervention helping to gain understanding of how NIDUS-Family influences change in goal attainment at a dyadic-level. Generation of themes and patterns will be used to test, develop, and refine the emerging theory that the values and approaches are important, in combination with the strategies, in the success of the intervention for dyads.

Trial Population-level data integration. A joint display[18] will be used to integrate the findings from the dyadic-level matrix with the trial population quantitative and qualitative acceptability questionnaire outcomes and trial data for dose, reach, fidelity, and attrition data. This will be used to draw inferences and interpret how NIDUS-Family works at a 'population' level. This approach will enable mapping of dyadic-level data to trial population data to identify the essential mechanisms of impact, implementation and contextual factors that influence change, in turn, refining and consolidating the emerging theoretical model of change for dyadic goal attainment and the NIDUS-Family logic model.

3. Ethics and dissemination

The NIDUS-Family trial which is funded by The Alzheimer's Society, has been registered on the clinical trials register at http://www.isrctn.com/ISRCTN11425138. NIDUS-Family ethics, which cover this evaluation have been approved with REC reference: 19/LO/1667, IRAS project ID: 271363.

The NIDUS study protocol includes a process evaluation section, an amendment was authorised in June 2021 to add additional qualitative interview processes with added facilitator and patient information sheets and consent forms, and to clarify that facilitators will also be interviewed. Changes to the acceptability questionnaire at 12 month follow up were also submitted and accepted.

Full details relating to ethics can be found in the NIDUS Study protocol][8]

The evaluation findings will be disseminated through publications and conferences. They will also inform recommendations for a future planned NIDUS-Family implementation study and strategy.

4. Discussion

This protocol follows the MRC guidance for process evaluations and outlines the rationale, design and methods for the process evaluation of the NIDUS-Family intervention. The focus of this evaluation is to identify the mechanisms of impact, implementation and contextual factors that influence goal attainment, and how these can help people with dementia live at home for longer. This evaluation is theory-driven and will test, develop, and refine the NIDUS-Family theory for goal attainment. Findings will be written up as recommendations that will feed into the NIDUS-Family implementation study.

Study Strengths. Following the MRC Guidance _for complex interventions provides a framework for planning, designing, conducting, analysing, and reporting a process evaluation. This framework provides a standardised approach to process evaluation enabling transparency across the relatively novel field of complex intervention process evaluation.

A convergent mixed-method design will combine qualitative and quantitative data iteratively to increase understanding of outcomes and improve the NIDUS-Family intervention for roll-out.[9,11]

For peer review only

At interpretation level, the two-phase approach initially uses a mixed-method matrix to triangulate qualitative and quantitative data at the dyadic level, then a joint display to integrate dyadic-level findings with trial population data. Each level will draw out new insights and interpretations leading to a deeper understanding of how NIDUS-Family influences change through goal attainment.[19] The mixed-methods design will allow for the emerging theory to be tested, refined, and developed to elicit the key mechanisms of impact, implementation and contextual factors that influence goal attainment through NIDUS-Family. These findings will be used to inform the implementation study rolling out NIDUS-Family into practice to maximise its impact in the 'real world'.

The evaluator is independent from the trial, albeit funded by the NIDUS Programme. To enable understanding and awareness of the trial the evaluator has access to the NIDUS programme team, enabling effective communication and responsiveness to process changes. The evaluation outcomes will not feed into the ongoing trial, they will however inform the post-trial implementation study.

As the NIDUS-Family trial is implemented across various geographical locations, with emphasis on recruiting a diverse population, this reduces the presence of cohort effect.

The evaluator will keep a reflexive journal throughout to capture methodological and theoretical decisions and, transparency and reflection on data collection, analysis, and integration.

Study limitations. There are some limitations to this protocol, firstly, data will only be collected at 12-month follow-up, for some dyads there may be a lag between finishing the intervention and evaluation.

Secondly, as the NIDUS-Family intervention is a complex model, this process evaluation will evaluate a small sample of participants. As there are many contextual factors (dementia severity, covid, local resources, dyadic relationships) the findings are taken at a specific point in time and account for the contexts relating to that specific dyad so may not be generalisable to difference contexts. The evaluation will reflect and represent the geographical and cultural diversity of the NIDUS-Family trial, this may not be fully reflective of the underlying population.

Finally, it is important to note that even though the facilitators' data will be anonymised, the facilitators are employed by the trial and are involved in trial data collection.

Author's Contribution: Protocol written by Danielle Wyman and it was critically edited for intellectual content by Professor Laurie Butler (ARU), Professor Claudia Cooper (UCL), Professor Peter Bright (ARU), Dr Sarah Morgan-Trimmer(Exeter) and Dr Julie Barber (UCL).

Funding Statement: This work was carried out within the UCL Alzheimer's Society Centre of Excellence for Independence at home, NIDUS (New Interventions in Dementia Study) programme (Alzheimer's Society Centre of Excellence grant 330).

Competing Interests Statement: The NIDUS-Family trial is funded by the Alzheimer's Society Centre of Excellence grant 330, which is led by CC, with LB as a co-investigator. DW is a PhD student funded by the grant.

For peer review only

References

1 Alzheimer's Society. Alzheimer's Society's view on demography. April 29 2021 . Date accessed November 19, 2020 URLhttps://www.alzheimers.org.uk/about-us/policy-and-influencing/what-we-think/demography

2 Wittenberg R, Hu B, Barraza-Araiza L et al. Projections of older people with dementia and costs of dementia care in the United Kingdom, 2019–2040. 2019. CPEC Working Paper 5. Accessed April 29 2021, URL https://www.alzheimers.org.uk/sites/default/files/2019-11/cpec_report_november_2019.pdf

3 Prince M, Knapp M, Guerchet M, et al. (2014). Dementia UK: Update. Date accessed November 19, 2020 from https://www.alzheimers.org.uk/sites/default/files/migrate/downloads/dementia_uk_update.pdf

4 Lord K, Livingston G, Robertson S, et al. (2016). How people with dementia and their families decide about moving to a care home and support their needs: development of a decision aid, a qualitative study. *BMC Geriatric* 2016;16:1

5 NICE Guideline [NG97]. (2018). Dementia: assessment, management and support for people living with dementia and their carers. Date accessed November 19, 2020 from https://www.nice.org.uk/guidance/ng97

6 England PH. Dementia: applying All our Health. London; 2018. Accessed November 19, 2020 URL https://www.gov.uk/government/publications/dementia-applying-all-our-health/dementia-applying-all-our-health

7 Lord K, Beresford-Dent J, Rapaport P, et al. Developing the New Interventions for independence in Dementia Study (NIDUS) theoretical model for supporting people to live well with dementia at home for longer: a systematic review of theoretical models and Randomised Controlled Trial evidence. *Social Psychiatry and Psychiatric Epidemiology;* 2020;55:1-14

8 Burton A, Rapaport P, Palomo M, Lord K, Budgett J, Barber J, Hunter R, Butler L, Vickerstaff J, Rockwood K, Ogden M, Smith D, Lang I, Livingston G, Dow B, Kales H, Manthorpe J, Walters K, Hoe J, Orgeta V, Samus Q, Cooper C; NIDUS study team. Clinical and cost-effectiveness of a New psychosocial intervention to support Independence in Dementia (NIDUS-family) for family carers and people living with dementia in their own homes: a randomised controlled trial. *Trials.* 2021 Dec 2;22(1):865. doi: 10.1186/s13063-021-05851-z. PMID: 34857029; PMCID: PMC8637036.

9 Moore G F, Audrey S, Barker M et al. Process evaluation of complex interventions: Medical Research Council guidance. *BMJ* 2015;350:1258

10 Tashakkori A, Teddlie C. Sage Handbook of Mixed Methods in Social and Behavioural Research. London: Sage 2010: 725-748

11 Creswell J W, Creswell J D. Research Design: Qualitative, Quantitative, and Mixed Methods Approaches. 5th Edition London: Sage 2018.

12 Moseholm E, Fetters M D. Conceptual models to guide integration during analysis in convergent mixed methods studies. *Sage Journals* 2017;10:2

13 Nelson J. Using conceptual depth criteria: addressing the challenge of reaching saturation in qualitative research. *Qualitative Research* 2016;17:

For peer review only

14 Braun V, Clarke V. Using thematic analysis in psychology. *Qualitative Research in Psychology* 2006;3:77-101

15 Braun V, Clarke V. *Successful qualitative research; a practical guide for beginners*. London: Sage 2013

16 Alwashmi M F, Hawboldt J, Davis E et al. The Iterative Convergent Design for Mobile Health Usability Testing: Mixed Methods Approach. *JMIR mHealth and uHealth* 2019;7:4

17 Wendler C. Triangulation using a meta-matrix. *Methodological Issues in Nursing Research* 2001;35:521-5

18 Guetterman T C, Fetters M D, Creswell J W. (2015). Integrating Quantitative and Qualitative Results in Health Science Mixed Methods Research Through Joint Displays. *Annals of Family medicine* 2015;13:6

19 Fetters M D, Curry L A, Creswell J W. Achieving integration in mixed methods designs principles and practices. *Health Services Research,* 2013;*48*:2134-2156

For peer review only

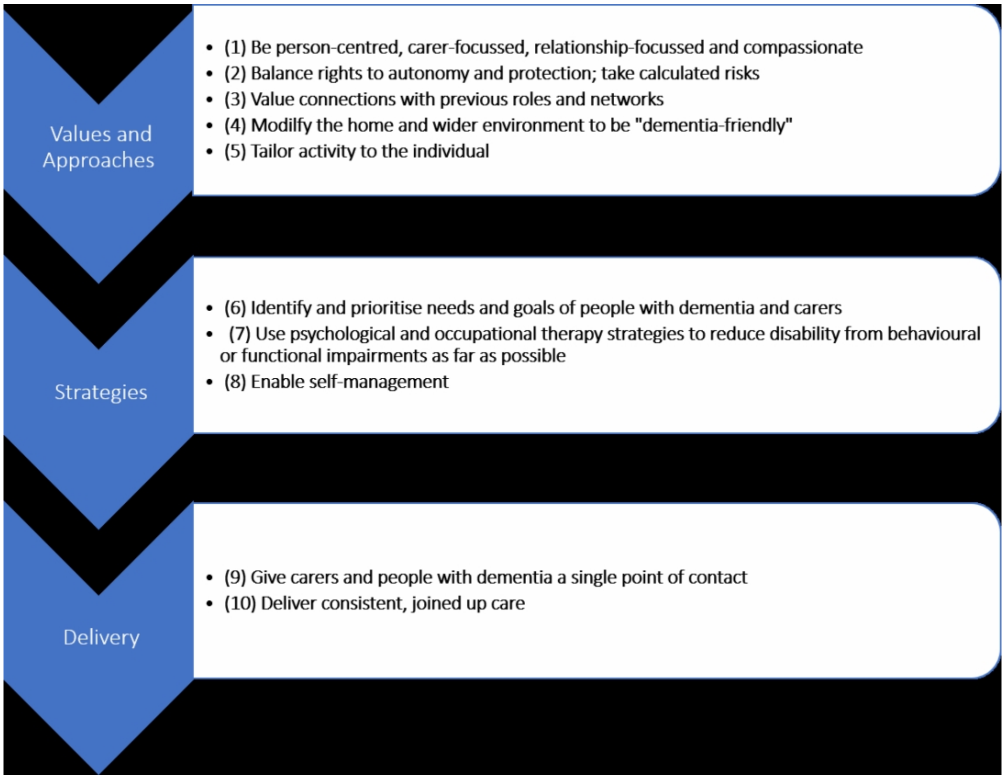

Figure 1. NIDUS Theoretical Model of Independence at Home

208x160mm (150 x 150 DPI)

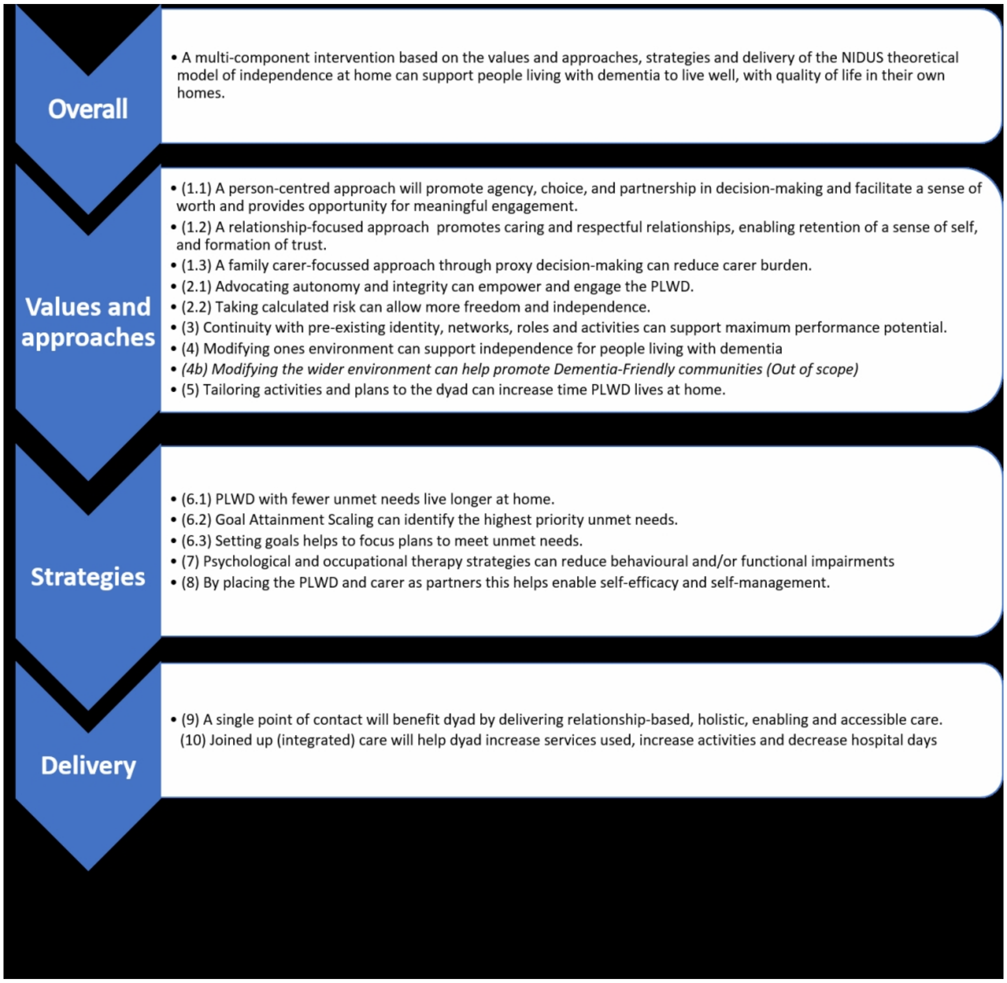

Figure 2.
Hypothesised NIDUS-Family Causal Assumptions

Note. Derived from NIDUS Theoretical Model of Independence at Home (Figure 1)

239x233mm (150 x 150 DPI)

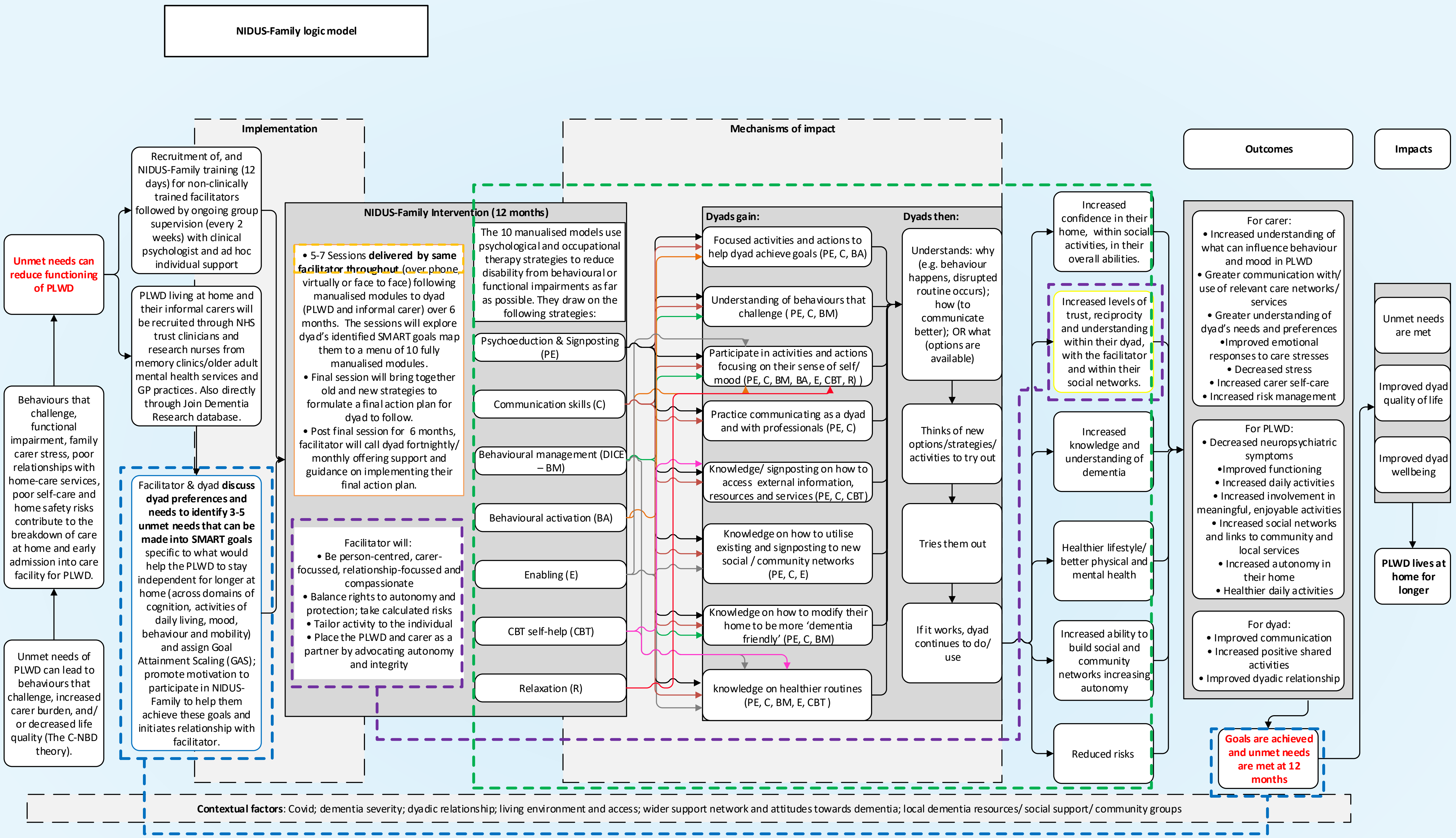
NIDUS-Family logic model
Unmet needs can reduce functioning of PLWD
Behaviours that challenge, functional impairment, family carer stress, poor self-care and relationships with home-care services, poor self-care and home safety risks contribute to the breakdown of care at home and early admission into care facility for PLWD.
Unmet needs of PLWD can lead to behaviours that challenge, increased carer burden, and/ or decreased life quality (The C-NBD theory).
Implementation
Recruitment of, and NIDUS-Family training (12 days) for non-clinically trained facilitators followed by ongoing group supervision (every 2 weeks) with clinical psychologist and ad hoc individual support
PLWD living at home and their informal carers will be recruited through NHS trust clinicians and research nurses from memory clinics/older adult mental health services and GP practices. Also directly through Join Dementia Research database.
Facilitator & dyad discuss dyad preferences and needs to identify 3-5 unmet needs that can be made into SMART goals specific to what would help the PLWD to stay independent for longer at home (across domains of cognition, activities of daily living, mood, behaviour and mobility) and assign Goal Attainment Scaling (GAS); promote motivation to participate in NIDUS-Family to help them achieve these goals and initiates relationship with facilitator.
NIDUS-Family Intervention (12 months)
• 5-7 Sessions delivered by same facilitator throughout (over phone, virtually or face to face) following manualised modules to dyad (PLWD and informal carer) over 6 months. The sessions will explore dyad's identified SMART goals map them to a menu of 10 fully manualised modules.
• Final session will bring together old and new strategies to formulate a final action plan for dyad to follow.
• Post final session for 6 months, facilitator will call dyad fortnightly/ monthly offering support and guidance on implementing their final action plan.
Facilitator will:
• Be person-centred, carer-focussed, relationship-focussed and compassionate
• Balance rights to autonomy and protection; take calculated risks
• Tailor activity to the individual
• Place the PLWD and carer as a partner by advocating autonomy and integrity
The 10 manualised models use psychological and occupational therapy strategies to reduce disability from behavioural or functional impairments as far as possible. They draw on the following strategies:
Psychoeduction & Signposting (PE)
Communication skills (C)
Behavioural management (DICE – BM)
Behavioural activation (BA)
Enabling (E)
CBT self-help (CBT)
Relaxation (R)
Mechanisms of impact
Dyads gain:
Focused activities and actions to help dyad achieve goals (PE, C, BA)
Understanding of behaviours that challenge (PE, C, BM)
Participate in activities and actions focusing on their sense of self/ mood (PE, C, BM, BA, E, CBT, R))
Practice communicating as a dyad and with professionals (PE, C)
Knowledge/ signposting on how to access external information, resources and services (PE, C, CBT)
Knowledge on how to utilise existing and signposting to new social / community networks (PE, C, E)
Knowledge on how to modify their home to be more 'dementia friendly' (PE, C, BM)
knowledge on healthier routines (PE, C, BM, E, CBT)
Dyads then:
Understands: why (e.g. behaviour happens, disrupted routine occurs); how (to communicate better); OR what (options are available)
Thinks of new options/strategies/ activities to try out
Tries them out
If it works, dyad continues to do/ use
Increased confidence in their home, within social activities, in their overall abilities.
Increased levels of trust, reciprocity and understanding within their dyad, with the facilitator and within their social networks.
Increased knowledge and understanding of dementia
Healthier lifestyle/ better physical and mental health
Increased ability to build social and community networks increasing autonomy
Reduced risks
Outcomes
For carer:
• Increased understanding of what can influence behaviour and mood in PLWD
• Greater communication with/ use of relevant care networks/ services
• Greater understanding of dyad's needs and preferences
• Improved emotional responses to care stresses
• Decreased stress
• Increased carer self-care
• Increased risk management
For PLWD:
• Decreased neuropsychiatric symptoms
• Improved functioning
• Increased daily activities
• Increased involvement in meaningful, enjoyable activities
• Increased social networks and links to community and local services
• Increased autonomy in their home
• Healthier daily activities
For dyad:
• Improved communication
• Increased positive shared activities
• Improved dyadic relationship
Goals are achieved and unmet needs are met at 12 months
Impacts
Unmet needs are met
Improved dyad quality of life
Improved dyad wellbeing
PLWD lives at home for longer
Contextual factors: Covid; dementia severity; dyadic relationship; living environment and access; wider support network and attitudes towards dementia; local dementia resources/ social support/ community groups

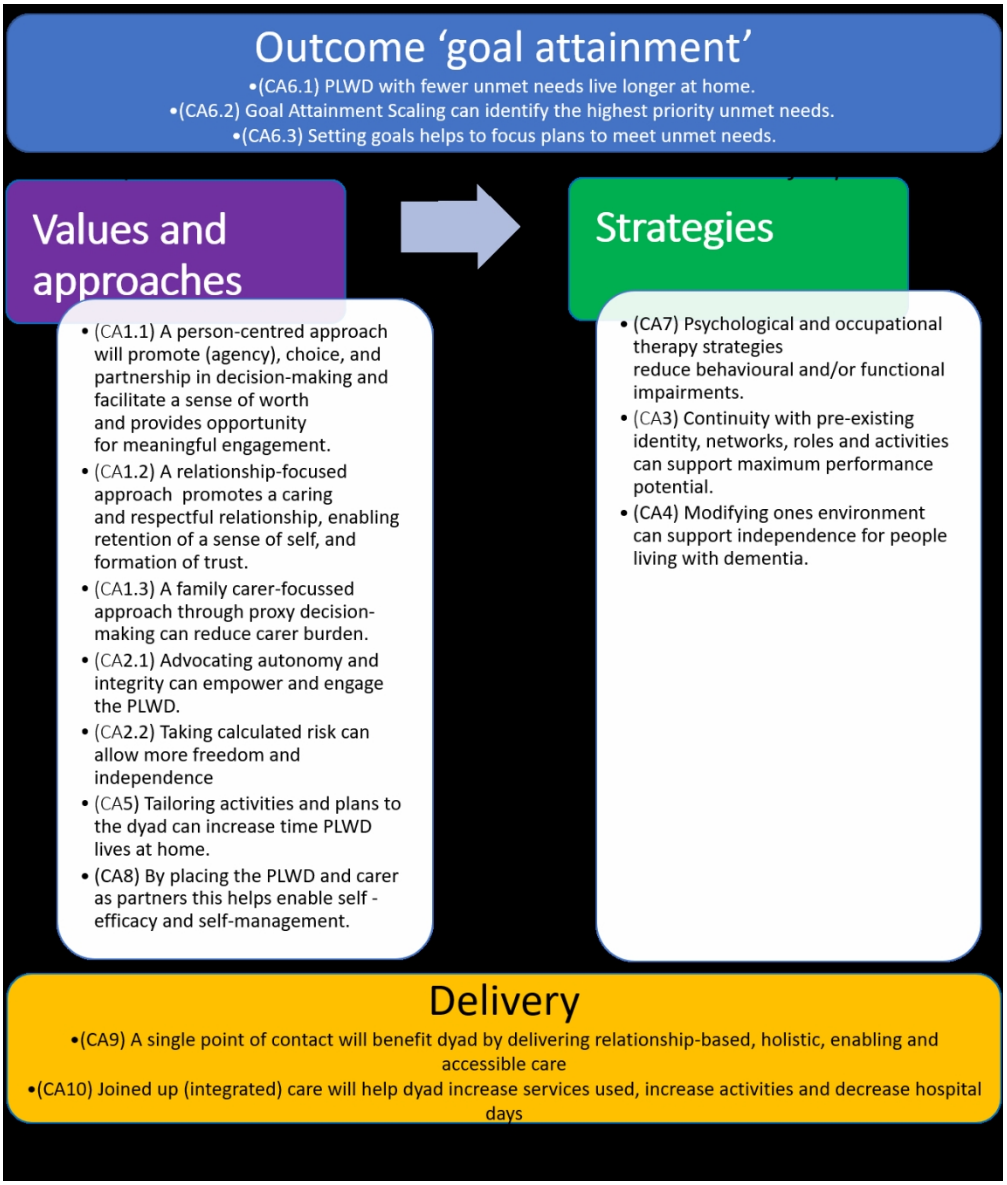

Figure 4.
Emerging Theoretical Model of Change for Attainment of Dyadic Goals through NIDUS-Family Intervention (with associated causal assumptions)

222x261mm (150 x 150 DPI)

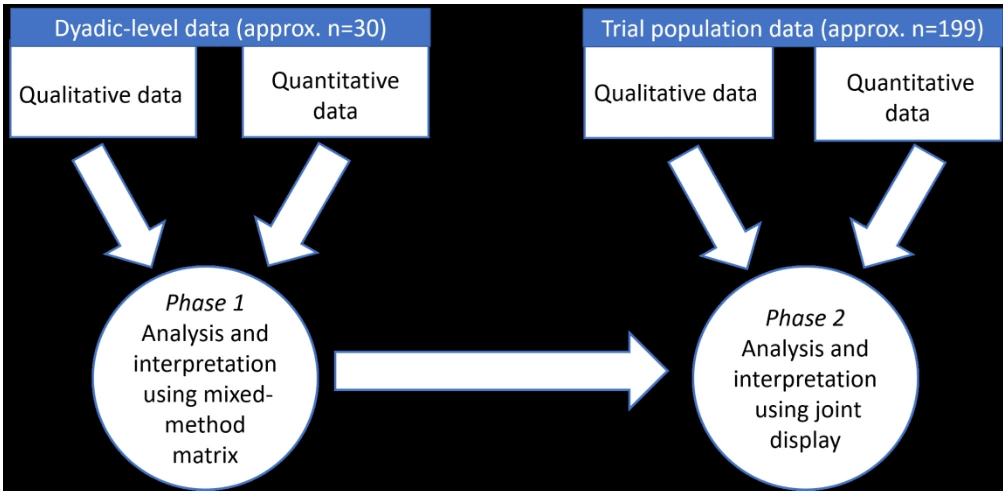

Figure 5.Mixed-Methods Convergent Design

388x189mm (150 x 150 DPI)

Appendix A

Alignment of planning, design and analysis of the NIDUS-Family process evaluation with MRC guidance.

Phase	MRC guideline recommendations (Moore et al, 2015)	Consideration of the recommendations for NIDUS-Family process evaluation
**Planning**	Define parameters of relationships of evaluators with intervention developers or implementers, balancing needs for good working relationships and independence; and how evaluators will inform implementation.	• Process evaluation led by a separate University.<br>• Evaluator is associate staff member at the trial University.<br>• NIDUS facilitators are employed through the intervention.<br>• Findings will inform the post-trial implementation strategy. They will not feed into the ongoing trial.
	Ensure the research team has the correct expertise, including, qualitative and quantitative research methods, and inter-disciplinary theoretical expertise.	Multi-disciplinary team includes expertise in psychology (ageing and behavioural change), old age psychiatry and dementia, neuropsychology, health service process evaluations, qualitative, quantitative, and mixed methods.
	Process and outcome evaluation team's degree of separation or integration:<br>• Oversight by a principal investigator.<br>• Good communication systems.<br>• Integration plans for process and outcome data agreed from the outset.	• Principal investigator has oversight over the NIDUS-Trial and is a subsidiary supervisor for evaluation lead.<br>•Evaluation is independent to the NIDUS-Family trial, but with weekly communication.<br>• Integration of process and outcome data will feed into the implementation study and strategy, but not into the trial.
**Designing**	Describe the intervention and its causal assumptions.	• The NIDUS-Family theory and causal assumptions are represented in a logic model (Figure 3).<br>• Section 1.1 describes the intervention, and 1.4 describes the causal assumptions
	• Identify questions by considering the intervention.<br>• Agree scientific and policy priority questions by considering the evidence for intervention assumptions.<br>• Consult with the evaluation team and policy/practice stakeholders.<br>• Identify previous process evaluations of similar interventions.	• The logic model informed the evaluation research questions.<br>• The multi-disciplinary team, including PPI, were consulted on the logic model.<br>• Relevant process evaluations were identified through a systematic review (PROSPERO ID: CRD42020221337).
	• Use quantitative methods to quantify key process variables and allow testing of pre-hypothesised	• Quantitative and qualitative methods will build upon one another to test, refine, and develop the NIDUS-Family

For peer review only

	mechanisms of impact and contextual moderators.<br>• Use qualitative methods to capture emerging changes in implementation, experiences of the intervention and unanticipated or complex causal pathways, and to generate new theory.<br>• Balance collection of data on key process variables from all sites or participants, with detailed case studies of purposively selected samples.<br>• Consider data collection at multiple time points to capture changes to the intervention over time.	logic model and emerging theory model (Figure 4).<br>• Quantitative methods will capture population level data on acceptability, reach, dose, attrition and secondary trial measures (approx. n=199). Quantitative observation data (approx. n=30) will enable detailed dyadic case-studies<br>• Qualitative interviews with purposively sampled dyads using GAS ratings (approx. N=30) will capture dyads experiences of receiving the intervention for case-studies and theme generation.<br>• Quantitative and qualitative methods will be matched on construct.<br>• Purposive sampling will recruit a sample representative of the trial population.<br>• Participants who withdraw will complete a questionnaire or an interview.<br>• Data collection at post 12-month follow-up for dyads and throughout for facilitators.
**Analysis**	Provide descriptive quantitative information on fidelity, dose and reach.	Fidelity: Fidelity checklist ratings for 20% of intervention-arm participants<br>Dose: number of sessions<br>Reach: Sites and locations<br>Attrition: Rate of withdrawal
	modelling of variations between participants or sites for factors such as fidelity or reach.	Contextual factors related to demographic data will be factored into data analysis and integration.
	Integrate quantitative process data into outcomes datasets, examining whether effects differ by implementation or pre-specified contextual moderators, and test hypothesised mediators.	Secondary trial data, dyadic observation fidelity checklist data, and acceptability ratings will be integrated to understand factors relating to high and low goal attainment.
	Collect and analyse qualitative data iteratively so that themes that emerge in early interviews can be explored in later ones.	Qualitative data collection and analysis will be carried out iteratively as dyads finish their 12-month follow-up. Emerging themes from earlier interviews will be explored in later interviews.
	quantitative and qualitative analyses build upon one another, with qualitative data used to explain	A two-stage integration approach will be used to merge the findings, initially at the

For peer review only

	quantitative findings, and quantitative data used to test hypotheses generated by qualitative data.	level of the dyad, then at the population level.
	Initially analyse and report qualitative process data prior to knowing trial outcomes to avoid biased interpretation.	Qualitative data will be collected and analysed before trial outcomes are known.
	Report whether process data are being used to generate hypotheses (analysis blind to trial outcomes), or for post-hoc explanation (analysis after trial outcomes are known).	Process data will be used to generate hypotheses, analysis will be blind to primary trial outcomes. Secondary outcomes will be analysed.

Note. Adapted from Moore et al (2015, p12)

For peer review only

Appendix B

Matching Quantitative and Qualitative Constructs Examples

**Construct**	**Associated causal assumptions**	**Quantitative questions** *Method: Acceptability questionnaire and observation data collected through listening to video/audio recordings of dyads session* *Variable: rate 1 Strongly disagree – 5 strongly agree)*		**Qualitative questions** *Method: Qualitative semi-structured interviews*	
		Acceptability questionnaire (Appendix F)	Observation checklist (Appendix E)	Dyad (Appendix C)	Facilitator (Appendix D)
Values and approaches	CA1.1		The facilitator promoted choice [for PLWD/ for Carer]	Do you feel you were able to contribute to the sessions?	Do you feel you promoted the dyad to have choice?
	CA1.1, 1.2, 8	[myself/ the person I care for] had a good relationship with my facilitator	Discussions were respectful/ supportive [for PLWD/ for Carer] *(allowing others to speak, actively listening, supporting their opinions, working as partners, discussing differing opinions calmly)*	• Do you feel you were respected?<br>•Do you feel you built up a level of trust with your facilitator?<br>• Do you feel the relationship was mutual and reciprocal?	• Do you feel you built a sense of trust with the dyad?<br>• Do you feel you actively listened?<br>• Do you feel there was mutual respect between you and the dyad?
	CA8		[PLWD/ Carer] had opportunities to ask questions.	How were your discussions in the sessions?	Who was involved in the discussion?
	CA1.3, 8	[I/ the person I care for] contributed to decision making.	[PLWD/ Carer] contributed to decision making.	Did you feel involved in the decision-making?	Who was involved in decision-making?

For peer review only

	CA1.1	[I/ the person I care for] had opportunities for meaningful engagement.	[PLWD/ Carer] had opportunities for meaningful engagement *(able to actively participate, actively contribute ideas, skills or abilities)*	Do you feel you were able to actively participate?	Do you feel the dyad had opportunities for meaningful engagement?
	CA2.1		[facilitator/Carer] showed compassion *(did they take time to bond, act with kindness, be encouraging, be polite)*	How did the facilitator make you feel?	How would you describe your persona in the sessions?
	CA2.2		[facilitator/PLWD/ Carer] explored risks [for PLWD/ for carer]	Did you discuss any possible risks?	Tell me about a risk you discussed and how you managed this (when setting tasks/ goals)?
	CA5	Goals were tailored to the [PLWD/ family member] needs.	The facilitator tailored [PLWD/ Carer] needs/goals/plans/activities/tasks.	• Do you feel the goals set reflected your needs/issues at the time?<br>• What plans, activities, tasks did you put in place to work towards your goals?	Once the goals were set can you talk through how you developed [plans/ activities/ actions] for the dyad to work towards their goals…
	CA1.3, 8		[facilitator/PLWD/ Carer] agreed (acknowledged) next steps (actions to follow the session)	Were you clear on activities between sessions?	*Who took accountability for actions?*
	CA8		The [PLWD/carer] acknowledged/ took ownership	Who took charge of doing the	Who took accountability for actions?

For peer review only

			for the actions/tasks s et.	activities/plan s?	
Goals and Strategies	CA6.3		Goals were discussed .	Tell me about your goals…	How did the [PLWD/carer] interact in the sessions?
	CA6.3	The modules helped [me/ the person I care for] work towards my goals…	Modules were discussed in line/ linked with the dyad's goals.	How did module [X] fit with/ affect your goals?	Talk me through how the modules worked for the dyad…
	CA6.3		[PLWD/ carer] engaged with the module/s…	How did you find the modules?	How do you feel the dyad engaged with the modules?
	CA6.3		Clear objectives/ next steps were set [for PLWD/Carer]	Who took charge of doing the activities/plan s?	Who took accountability for actions?

For peer review only

Appendix C

Semi-structured interviews for dyads

Interview Approach

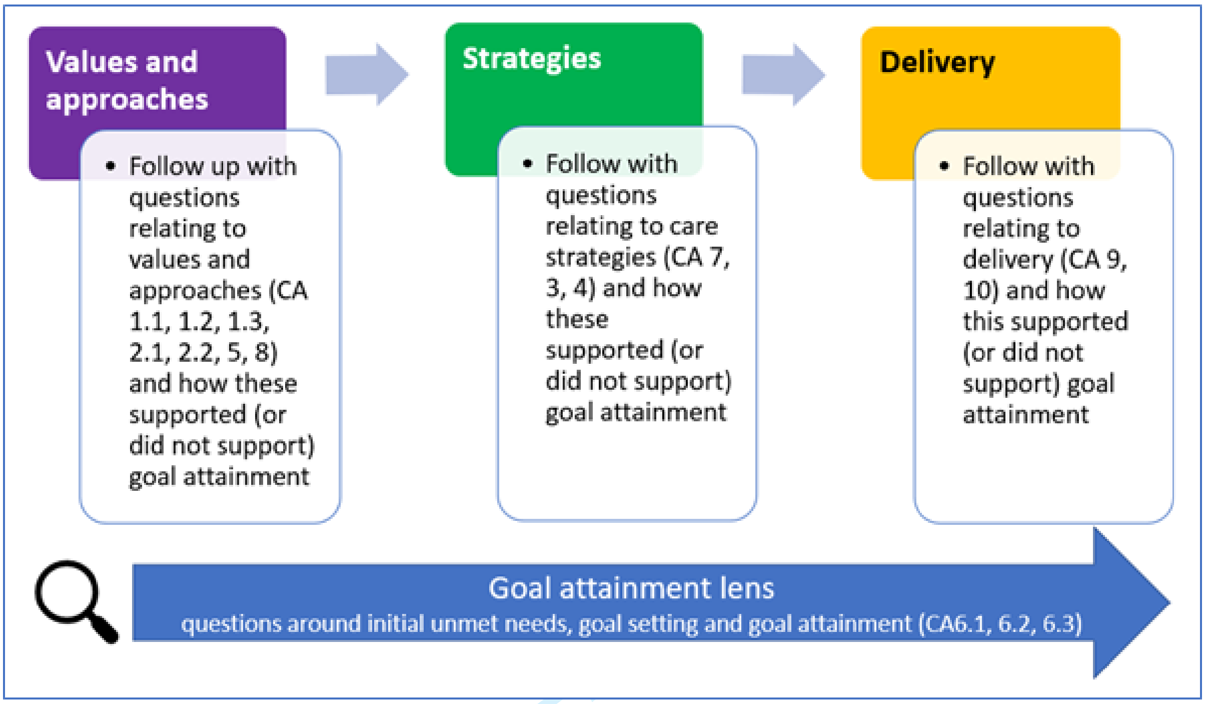

Semi-structured topic guide for dyads (PLWD and carer)

This will be used as a guide, a flexible approach will be adapted to capture any relevant information as well as unanticipated or unexpected pathways.

Introduction:

"Hello, I am XXXX and my role is XXXX. Thank you for your time today, I understand you have taken part in the NIDUS-Family intervention and I wanted to find out more about your experiences in receiving this. It would be interesting to explore your experiences of the NIDUS-Family trial, particularly in relation to your goals. There are no right or wrong answers, you are the experts. This interview will be audio-recorded and transcribed anonymously. Everything you say will be confidential and anonymised, any identifying information will be removed. You will be able to withdraw from this part of the study until the point of data analysis. Information from today will help us make sure any benefits of NIDUS-Family can be put into wider practice. Do you have any questions? If you are happy to continue, please can you give your consent (informed or by proxy) and I will start recording.".

Q1. Tell me about your experiences of NIDUS-Family… (where relevant ask PLWD first, then carer)

Prompts:

- How did you get involved with NIDUS-Family?
- What were your expectations of taking part in NIDUS-Family?

For peer review only - http://bmjopen.bmj.com/site/about/guidelines.xhtml

Q2. Tell me about the main things that have changed for you since finishing the NIDUS-Family intervention… *[ask PLWD and carer separately where possible]*

- How has that affected your day-to-day routine?
- How has that affected you?

Values and approaches

Q3. Tell me about your (PLWD and Carer) relationship?

- Overall:
  - *Day to day examples*
  - *Do you live together/ how often do you see each other?*
  - *Nature of visits?*
- NIDUS-Family specific
  - *How do you feel you worked together during the NIDUS sessions?*
  - *How were your discussions in the sessions?*
  - *Has your relationship changed since NIDUS-Family?*

Q4. Tell me about your relationship with the facilitator… (where relevant ask PLWD first, then carer)

  - *How did the facilitator make you feel?*
  - *Do you feel you were able to actively participate?*
  - *Do you feel you were able to contribute (ideas) to the sessions?*
  - *Did you feel involved in the decision-making?*
  - *Do you feel you built up a level of trust with your facilitator?*
  - *Do you feel the relationship was mutual and reciprocal?*
  - *Did you feel you partnered with the facilitator?*
  - *Do you feel you were respected?*
- Tell me how your relationship with your facilitator affected you attaining your goals?
- How did you feel about having the same facilitator throughout?

Q5. Tell me about your support network...

- informal – neighbours, friends, other family
- formal – services, resources
  - *Has this changed since starting/ finishing NIDUS-Family?*
  - *How did these (support/network/service use) changes affect your goals?*

Goal attainment

Q6. Tell me about your goals… (where relevant ask PLWD first, then carer)

Prompts:

- Do you feel the goals set reflected your needs/issues at the time?
  - *How did you discuss your needs?*
  - *Did you talk about your issues/needs?*
  - *Were they specific to you [PLWD and/or Carer]*
- Tell me more about how you decided on these goals?
  - *How were the goals set?*
  - *Who was involved in setting them?*
  - *How did that discussion go? Maybe we could use an example…*
- Do you feel your goals were achievable?
  - *[If not] Did you tell the facilitator you felt this at the time?*

For peer review only

        - *Is this in hindsight?*
    - Once you set your goals, tell me what you did next?
        - *How did you work towards your goals?*
        - *What plans, activities did you put in place?*
        - *Who came up with those plans?*
        - *Who took charge of doing the activites/plans?*
        - *Can you give me an example of something that helped?*
        - *Can you give me an example of something that may have got in your way?*
    - Could you tell me why you scored your goals as (+2/ 0,-1,-2)?
    - Knowing what you do now, is there anything you would have done differently?

Strategies

Q7. How did you find the modules (name modules *talk through one at a time*)?

- How did module [X] fit with your goals?
- How did module [X] affect your goals?
    - *What did you enjoy?*
    - *What did you not enjoy?*
- Were there any parts of the modules you liked?
- Were there any parts of the modules you didn't like?
- Is there anything you wish the modules had covered?

Q8. Before we finish, can I review the key points you mentioned about:

- Goal attainment
- Values and approaches
- Strategies
- Delivery

Q9. Is there anything else you would like to add?

Q10. Is there anything we haven't covered you feel is important?

Thank you for your time and for taking part today.

For peer review only

Appendix D

Semi-structured topic guide for NIDUS-Family facilitators

Interview approach

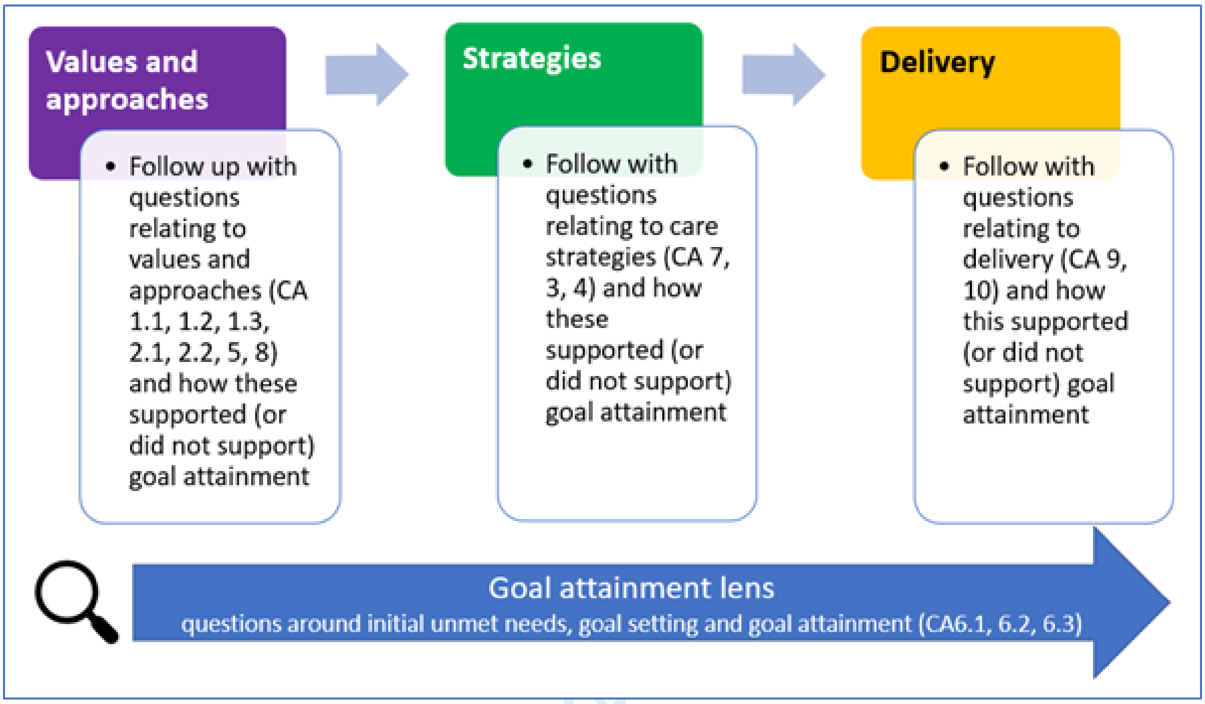

This will be used as a guide. A flexible approach will be adapted to capture any relevant information as well as unanticipated or unexpected pathways.

<u>Topic guide for facilitators</u>

Introduction:

"Hello, I am XXXX and my role is XXXX. Thank you for your time today, I wanted to find out more about your experiences in delivering the NIDUS-Family intervention. It would be interesting to explore things that you found went well and things that you feel could be improved to help us plan how this can be delivered in practice. There are no right or wrong answers, I'm interested in your experiences. This interview will be audio-recorded and transcribed, with your permission. Everything you say will be confidential and anonymised. Do you have any questions? If you are happy to continue, I will start recording.".

Q1. Can you tell me about the NIDUS-Family intervention?

- What are your experiences of the programme so far?
  - *Anything that was helpful?*
  - *Were there times when you felt uncomfortable delivering it?*
  - *Were there times the dyad felt uncomfortable receiving it?*

Q2. Can you tell me about your training?

- *How did you feel about delivering your first session?*
- *Can you give me an example…*

- Can you tell me what the top 'take homes' or 'key messages' you remember were from your training?
- Knowing what you do now, do you feel your training prepared you for your first session?
- Can you tell me about the support/ supervision you received through both training and when delivering?
  - *Anything that was helpful – can you give me an example?*
  - *What did you discuss with your supervision team – can you give me an example?*

<u>Values and approaches</u>

Q3. Can you tell me more about your relationship with dyad [XXX] across the sessions?

- *Do you feel you promoted the dyad to have choice (CA1.1)?*
- *Do you feel you promoted the dyad to have active agency? (CA1.2)*
- *Do you feel you built a sense of trust with the dyad? (CA1.2)*
- *Do you feel you actively listened?*
- *Do you feel your relationship provided mutuality and reciprocity? (CA1.2)*
- *Do you feel there was mutual respect between you and the dyad?*

- How do you feel your relationship affected the dyad attaining their goals?
- Do you feel the dyad had opportunities for meaningful engagement (able to actively participate, actively contribute ideas/ skills/abilities)?
- Can you tell me about the 'role' you played (parent-child relationship/ parent-parent relationship with dyad)?

Q4. Can you tell me about the dyads' relationship?

- *How did the dyad interact in the sessions?*
- *Who was involved in discussions?*
- *Who was involved in decision-making?*
- *Who took accountability for actions?*

- Expanding on the dyads' relationship – Can you give me an example of where the PLWD led on a suggestion or idea?

<u>Goal attainment</u>

Q5. Tell me about your experience of identifying issues/ needs with dyad XXXs…

- *How did you discuss this?*
- *Who led this conversation?*
- *Who identified the issues?*
- *How did dyads interact?*
- *Did dyads agree?*

- How were dyad [XXX] goals related to – PLWD/Carer or both?
- After you identified the dyad XXX '<u>needs</u>' tell me how you went about setting their goals…
  - *If it is easier, talk me through a specific example.*
  - *How did you link the need to their goal?*
  - *How was the dyad involved in the discussion?*

For peer review only

- *Was it PLWD and carer? Tell me more…*

Q6. Once the goals were set can you talk through how you developed [plans/ activities/ actions] for the dyad to work towards their goals…

- How were the dyad involved?
- Tell me about a risk you discussed and how you managed this (when setting tasks/ goals)?
- [If relevant] Dyad XXX rated themselves as [+2/ 0,-1,-2] could you tell me your views on why?
- [If relevant] The outcome assessor rated dyad [XXX] as [?] at 12-month follow up – do you agree? What would you have rated them?

Strategies

Q7. Talk me through how the modules worked for dyad [XXX]?

- What was your experience of delivering the manualised modules?
- How did you align dyad [XXX] goals to the modules?
- How do you feel the dyad [XXX] engaged with the modules?
- Can you give me an example where the modules helped motivate dyad [XXX]?
- Can you give me an example where the dyad did not understand or didn't 'click' with the module?
- *[if relevant]* Did you have discussions around adapting their home?
- *[if relevant]* Tell me about how adaptations to the dyads home affected them achieving their goals?

Delivery

Q8. What are your views on having one facilitator for each dyad?

To conclude

Q9. Before we finish, can I review the key points you mentioned about:

- **Goal attainment**
- **Values and approaches**
- **Strategies**

Q10. Is there anything else you would like to add?

Q11. Is there anything we have not covered you feel is important?

Thank you for your time and for taking part today.

For peer review only

Appendix E

Observation/ Fidelity Checklist

For fidelity checklist only complete italicised sections. Complete all sections for process evaluation observations.

Process evaluation factors	Please rate<br>1 Strongly disagree, 2 disagree, 3 neither agree nor disagree, 4 agree, 5 strongly agree	Examples – include descriptive text or quotations to demonstrate related observations<br>*(Free text)*
**Values and approaches (CA1.1, 1.2, 1.3, 2.1, 2.2, 5, 8)**		
[facilitator/PLWD/ Carer] paid attention (are they focused) in the session. [Fidelity checklist]	*For PLWD:*<br>*For Carer:*<br>*Facilitator:*	
**[facilitator/PLWD/ Carer] were engaged (actively contributed, followed the discussions) in the session.**	For PLWD:<br>For Carer:<br>Facilitator:	
**[facilitator/PLWD/ Carer] contributed to discussions.**	For PLWD:<br>For Carer:<br>Facilitator:	
**The facilitator promoted choice (CA1.1).**	For PLWD:<br>For Carer:<br>Overall:	
**Discussions were respectful (allowing others to speak, supporting their opinions, working as partners, discussing differing opinions calmly - CA1.1, 1.2 8).**	Between PLWD and carer:<br>Between PLWD and facilitator:<br>Between carer and facilitator:<br>Overall	
**[PLWD/ Carer] had opportunities to ask questions.**	PLWD:<br>Carer:	
**[PLWD/ Carer] contributed to decision making (CA1.3)**	For PLWD:<br>For Carer:<br>Overall:	
**[PLWD/ Carer] had opportunities for meaningful engagement (able to actively participate, actively contribute ideas, skills or abilities) for PLWD (CA1.1)**	For PLWD:<br>For Carer:	
**[facilitator/Carer] showed compassion (did they take time to bond, act with kindness, be encouraging, be polite - CA2.1)?**	Facilitator to PLWD:<br>Carer to PLWD:<br>Facilitator to carer:	
**[facilitator/PLWD/ Carer] explored risks (CA2.2).**	For PLWD:<br>For Carer:<br>For facilitator:<br>Overall:	

For peer review only

**the facilitator tailored [PLWD/ Carer] needs/goals/plans/activities/tasks (CA8).**	For PLWD:<br>For Carer:<br>Overall:	Needs:<br>Goals:<br>Plans:<br>Activities:<br>Tasks:
**[facilitator/PLWD/ Carer] agreed (acknowledged) next steps (actions to follow the session).**	PLWD:<br>Carer:<br>Facilitator:	
**Did the [PLWD/carer] acknowledge/ take ownership for the actions/tasks set?**	PLWD:<br>Carer:	
**Goals (CA6.1, 6.2, 6.3) and Strategies (CA7, 3, 4)**		
**Goals were discussed.**		Which goals?
**Modules were discussed in line/ linked with the dyad's goals.**		Which modules?
**Clear objectives/ next steps were set [for PLWD/Carer].**	For PLWD:<br>For carer	
**The facilitator kept the [PLWD/Carer] focused on the module/goal.**		
**Overall**		
**The group was relaxed.**		
**The facilitator kept the [PLWD/carer] engaged in the session.**	PLWD:<br>Carer	
**The facilitator kept the [PLWD/ carer] focused on the manual/goals.**	PLWD:<br>Carer:	
**The facilitator kept the session to time.**		

**Any additional notes on the relationship dynamics between the:**<br>**• Facilitator and PLWD**<br>**• Facilitator and carer**<br>**• PLWD and carer (Free text)**

For peer review only

**Any additional notes on the session (Free text)**
Any additional notes on impact of COVID-19 on delivery (Free text) [Fidelity checklist]
Any additional notes for modifications to the intervention or facilitator training (Free text) [Fidelity checklist]

For peer review only - http://bmjopen.bmj.com/site/about/guidelines.xhtml

APPENDIX F

Acceptability Questionnaire for Family Carers

Family carer ID ------------------------ Person living with dementia ID: -------------------------

GAS scores: --------------------------------

NIDUS-Family acceptability scale: Family carer (Please tick the box you feel is the most relevant answer related to the question, rating from strongly disagree to strongly agree)

Statement		Strongly disagree	Disagree	Neither agree nor disagree	Agree	Strongly agree
The intervention helped the person I care for.						
[I/ the person I care for] contributed to decision-making.	Myself (family carer)					
	The person I care for					
[I/ the person I care for] had opportunities for meaningful engagement *(able to actively participate, actively contribute ideas, skills or abilities)*	Myself (family carer)					
	The person I care for					
Goals were tailored to [my/ the person I care for] needs.	Myself (family carer)					
	The person I care for					
The modules helped [me/ the person I care for] work towards my goals.	Myself (family carer)					
	The person I care for					
[myself/ the person I care for] had a good relationship with my facilitator.	Myself (family carer)					
	The person I care for					
The intervention helped improve my relationship with the person I care for.						

What feedback do you have for us about your experiences of receiving the NIDUS-Family intervention?

....

.... ***[Please turn over for more space]***

For peer review only

For peer review only

Appendix G

Withdrawal Questionnaire

Family carer ID ------------------------

Person living with dementia ID: ---------------------------

**What was the reason(s) you withdrew?**
**Would you do anything differently?**
**What would you change?**

For peer review only

**Any other comments**

For peer review only
